# Long-term breeding progress of yield, yield-related, and disease resistance traits in five cereal crops of German variety trials

**DOI:** 10.1007/s00122-021-03929-5

**Published:** 2021-10-15

**Authors:** F. Laidig, T. Feike, B. Klocke, J. Macholdt, T. Miedaner, D. Rentel, H. P. Piepho

**Affiliations:** 1 Biostatistics Unit, Institute of Crop Science, University of Hohenheim, Fruwirthstrasse 23, 70599 Stuttgart, Germany; 2 Julius Kühn Institute (JKI), Federal Research Centre for Cultivated Plants, Institute for Strategies and Technology Assessment, Stahnsdorfer Damm 81, 14532 Kleinmachnow, Germany; 3 Institute of Plant Breeding I and Agronomy, Justus-Liebig-University Giessen, Schubertstrasse 81, 35392 Giessen, Germany; 4 Department of Plant and Environmental Sciences, Section of Environmental Chemistry and Physics, University of Copenhagen, 1871 Frederiksberg, Copenhagen, Denmark; 5 State Plant Breeding Institute, University of Hohenheim, Fruwirthstrasse 21, 70599 Stuttgart, Germany; 6 Bundessortenamt, Osterfelddamm 60, 30627 Hannover, Germany

## Abstract

**Key message:**

Considerable breeding progress in cereal and disease resistances, but not in stem stability was found. Ageing effects decreased yield and increased disease susceptibility indicating that new varieties are constantly needed.

**Abstract:**

Plant breeding and improved crop management generated considerable progress in cereal performance over the last decades. Climate change, as well as the political and social demand for more environmentally friendly production, require ongoing breeding progress. This study quantified long-term trends for breeding progress and ageing effects of yield, yield-related traits, and disease resistance traits from German variety trials for five cereal crops with a broad spectrum of genotypes. The varieties were grown over a wide range of environmental conditions during 1988–2019 under two intensity levels, without (I1) and with (I2) fungicides and growth regulators. Breeding progress regarding yield increase was the highest in winter barley followed by winter rye hybrid and the lowest in winter rye population varieties. Yield gaps between I2 and I1 widened for barleys, while they shrank for the other crops. A notable decrease in stem stability became apparent in I1 in most crops, while for diseases generally a decrasing susceptibility was found, especially for mildew, brown rust, scald, and dwarf leaf rust. The reduction in disease susceptibility in I2 (treated) was considerably higher than in I1. Our results revealed that yield performance and disease resistance of varieties were subject to considerable ageing effects, reducing yield and increasing disease susceptibility. Nevertheless, we quantified notable achievements in breeding progress for most disease resistances. This study indicated an urgent and continues need for new improved varieties, not only to combat ageing effects and generate higher yield potential, but also to offset future reduction in plant protection intensity.

**Supplementary Information:**

The online version contains supplementary material available at 10.1007/s00122-021-03929-5.

## Introduction

Cereals are the most grown crops in Europe. They covered 53.8% of annual crops in the EU-28 in 2020 (https://ec.europa.eu/info/news/eu-agricultural-outlook-arable-land-area-continue-its-decline_en). Also in Germany, more than 50% (2014–2018) of total arable land is cultivated with cereals (StatJ 2019). In the German market, 29% of cereal grain is used for human food, 44% for livestock feeding, 13% for industrial uses, 10% for bioenergy, and 2% for seed (BLE 2019). Breeding progress has substantially increased potential yields of cereals in Germany by about 0.6%–1.3% per year in variety trials over the last 30 years, while on-farm yields only increased by about 0.5%–1%, leading to increasing yield gaps (Laidig et al. 2014). In this regard, insufficiently controlled fungal pathogens are one of the reasons for hampering higher on-farm yield progress.

Despite intensive crop protection measures and progress in resistance breeding, considerable yield losses for cereals due to diseases and lodging are reported by numerous studies (e.g. Jaysena et al. 2007; Wiik 2009; Jahn et al. 2012; Fones and Gurr 2015; Jevtic et al. 2017; Laidig et al. 2021; Willocquet et al. 2021). Oerke and Dehne (2004) reported yield losses due to pathogens of 10% in wheat and 14% in barley in Northwest Europe.

While most of the studies evaluated yield loss due to diseases, we found only few studies evaluating the breeding progress for disease resistance and lodging. Most of them focused on winter wheat (Ahlemeyer and Friedt 2011; Leisova-Svobodova et al. 2020; Voss-Fels et al. 2019; Zetsche et al. 2020; Zhang et al. 2020), a few on barley (e.g. Grausgruber et al. 2002; Ahlemeyer et al. 2008), one on triticale (Losert et al. 2017) but none on rye. Only few studies on breeding progress built on historic variety trial time series data (e.g. Mackay et al. 2011; Laidig et al. 2021). Most studies were conducted using historic varieties released over a period of several decades and growing them over 2 to 3 years under natural infection (e.g. Losert et al. 2017; Ahlemeyer et al. 2008; Berry et al. 2015) or artificial inoculation (e.g. Zetsche et al. 2020).

In this regard, one needs to note that there is a major difference in assessing the breeding progress of diseases from trials with historic varieties (also called ‘vintage trials’) and breeding progress based on historic data. In general, results from trials with historic varieties showed a stronger improvement of disease resistance compared to the progress assessed by historic data (Laidig et al. 2021). This difference can be ascribed to the dynamic virulence of biotrophic pathogen populations. Varieties tend to partially or fully lose their resistance against specific pathogens with increasing age. In extreme cases, the resistance against a specific disease can break down completely (Mackay et al. 2011), as was the case for yellow rust in some cultivars due to the advent of the aggressive Warrior race (Hovmøller et al. 2016). Piepho et al. (2014) described the quantitative assessment of loss of resistance with increasing variety age as “age effect”.

Breeding towards varieties with higher resistance against biotic stress is gaining more and more importance (Figueroa et al. 2018; Audenaert et al. 2014; Singh et al. 2019; Miedaner and Wilde 2019). On the one hand, climate change is expected to alter the relative importance of the fungal diseases strongly (Miedaner and Juroszek 2021). On the other hand, the global demand for agricultural products is continuously increasing, while the public debate on the use of pesticides and the negative environmental impact of intensive crop production in the industrial countries is increasing. Therefore, it is a persistent challenge to realize higher yields with less negative environmental impact, i.e. a sustainable intensification of crop production. It is an important goal of agricultural policies in the European Union, including Germany, to reduce the surplus of nitrogen fertilizer and the application of pesticides to mitigate the negative environmental impact and improve the sustainability of plant production (BMEL 2019; EU 2019). The European Commission aims to reduce the overall use of chemical pesticides and especially the use of more hazardous pesticides by 50% by 2030 (EU 2020), which will most likely go along with a continuous reduction of available effective plant protection compounds.

Against this background, new improved varieties are one of the most important and economically favourable resources to cope with the demand for increased food production, for a growing world population and the challenges of climatic change and the agro-political goals for a more sustainable plant production. Numerous studies on breeding progress for higher yield and improved resistance against biotic and abiotic stress have been reported as described above, mostly for winter wheat, but only few for other cereal crops. To the best of our knowledge, so far, no long-term study was published that quantified and compared long-term trends in breeding progress and ageing effects for cereal crops over that many agronomical traits, diseases, years, sites, and genotypes under such a wide range of pedo-climatic conditions as included in this study.

We provide a comprehensive overview of the long-term breeding progress and ageing effects of yield, yield-related and disease resistance traits from five cereal crops based on data from official variety trials carried out across Germany from 1988 to 2019 under two treatment intensities. In particular, we investigated (i) the trends in yield-related parameter, (ii) the trends for ageing effects of varieties, (iii) the genotypic and environmental variation and (iv) we compared the results across crops.

## Materials and methods

### Cereal variety trials

**Fig. 1:**
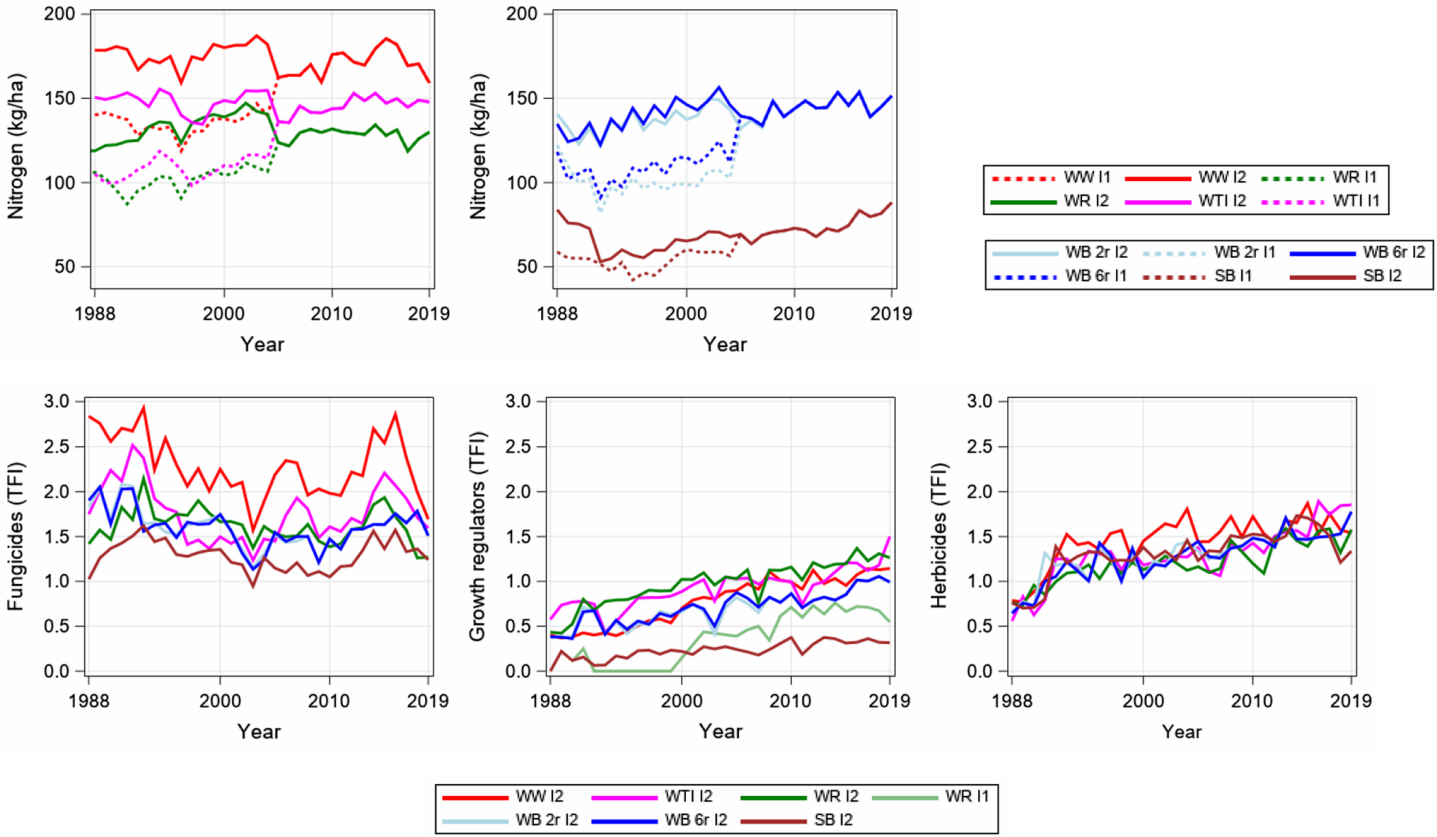
Applied average nitrogen rate in intensity 1 (I1) and 2 (I2) and treatment frequency index (TFI) for herbicides applied to I1 and I2, fungicides and growth regulators applied in I2 and for WR in I1. *WW* Winter wheat, *WTI* Winter triticale; *WR* Winter rye; *WB* Winter barley, *2r* two-row, *6r* six row varieties; *SB* Spring barley

The study was based on data from official variety trials for cereal crops carried out across Germany from 1988 to 2019 under two treatment intensities. The underlying variety testing procedure will be explained in the following. In the European Union, newly bred genotypes have to be officially tested for their value for cultivation and use (VCU) before they can be released for commercial use. In Germany, all cereal crops are tested by the Federal Office of Plant Varieties (German Bundessortenamt; bundesortenamt.de) for three years at multiple locations covering all pedo-climatic conditions of their typical growing regions. Additionally, at least three reference varieties are included in each series, which are identical over sites and within each series. Well-established varieties are chosen as references, representing the actual state of breeding progress. The references are updated on a regular basis, ensuring at least partial overlap of sets of references used in successive years. For the period before 1990, only data from West German locations are available. After each trial year, varieties, which do not prove additional VCU, are withdrawn from the series. In consequence, only about one third to one fourth of the varieties that originally entered the testing scheme, will be finally tested over the entire three years. Trials are conducted with up to three intensities of different application rates for nitrogen, fungicides and growth regulators, where intensity 1 (hereinafter referred to as I1) generally receives no fungicides or growth regulators and less nitrogen; only in winter rye growth regulators are also applied in I1, but at a lower rate than in intensity 2 (hereinafter referred to as I2). From 1991 onwards, varieties are tested with only two intensities. From 2005 onwards, the nitrogen application rates were standardized and both intensities received the same nitrogen rates (See Fig. 1). In order to be able to consider two intensities in every year over the entire time series, we averaged data of intensity 2 and intensity 3 for the period 1988 to 1990 and used this average as intensity 2. Herbicides and insecticides are applied for all intensities at the same level according to demand. In I2, fungicide application, fungicide selection and application rate are based on the average disease severity of the majority of varieties. The basis for treatment is thus neither the most susceptible nor the most resistant variety. Good local agricultural practice is used for treatment of ears, which are often not yet showing symptoms. Weather conditions are much more decisive for the timing of treatment. Trials are laid out in split-plot designs with main plots arranged in complete blocks. The treatments are applied to main plots, and the varieties are arranged in subplots. Subplots within main plots are either laid out as randomized blocks, or as alpha-lattice designs. The harvested average plot size is about 10 m^2^. Winter barley two-row and six-row varieties are grown in separate trials until 2006 and afterwards together in the same trial. Nevertheless, we considered two- and six-rowed types from 1988 on as grown in separate trials. Winter rye hybrid and population varieties are grown in the same trial and also treated identically during 1988–2019. However, we analysed both types as separate trials.

We evaluated data over a time period of 32 years from 1988–2019 for five cereal crops, i.e. winter wheat (WW), winter rye hybrid (WR Hyb) and population (WR Pop) varieties, winter triticale (WTI), winter barley (WB) two-rowed (2r) and six-rowed (6r) varieties and spring barley (SB). Varieties in all crops were line varieties except for WR. These crops account for 48% (2015–2019) of total arable land in Germany. WW is the most important crop (26%), followed by WB (11%), WR (5%), WTI and SB, with 3% each. For WTI, the first separate trial series started in 1988. To have a comparable time period in all crops, we started the entire study in this year.

In each crop, we assessed all traits for I1 and I2 as described in Table 1. Grain yield (YLD), ear density (EAD), single ear weight (EAW), plant height (HGT), days from sowing to ear emergence (EAE), lodging (LDG) and powdery mildew (MLD) were available in each crop. The other traits were crop-specific: in WW brown (leaf) rust (BNR), Septoria leaf blotch (Septoria tritici blotch, STB), Septoria nodorum blotch (SNB), yellow (stripe) rust (YLR) and tan spot (DTR); in WR stem buckling (SBL), BNR and Rhynchosporium (RYS); in WTI BNR, STB, YLR and RYS; in WB and SB SBL, ear buckling (EBL), RYS, net blotch (NTB) and dwarf leaf rust (DLR). Pathology for lodging and disease traits were described in Supplementary Material SM1; for more detailed information on cereal diseases see, e.g. AHB (2020) and Bailey et al. (2003). All evaluated traits were considered important for determining VCU and for the description of released varieties in the annually published “Descriptive Variety List” (BSL 2020). In the early years of the data used in this study, both Septoria diseases were estimated in trials as one joint rating, because the visual symptoms were hard to differentiate. Later on, STB became predominant while SNB lost its importance in leaf and ear infections.

**Table 1:** Description of traits for major cereals investigated in German variety trials during 1988–2019

Trait	Unit	Code	Description / Causal agent	EPPO code
*Continuous traits*				
Grain yield	dt ha^−1^	YLD	Grain yield at 86% dry matter	
Number of ears per m^2^	m^−2^	EAD	Number of ear bearing stems after ear emergence	
Single ear yield	g	EAY	Grain weight of a single ear	
Plant height	cm	HGT	Plant height after growth termination	
Ear emergence	days	EAE	Days from sowing to ear emergence	
*Score traits*				
*Stem stability*				
Lodging before harvest		LDG	Canopy is permanently displaced from the vertical	
Stem buckling (Culm buckling)		SBL	Stem buckles below the upper third part of the stem	
Ear buckling		EBL	Ear buckles in the upper third part of the stem	
*Diseases*				
Powdery mildew		MLD	*Blumeria graminis* f. spp. (formerly, *Erysiphe graminis* f.spp.)	ERYSGT
Brown rust (Leaf rust)	1–9	BNR	*Puccinia triticina* (*Puccinia recondita* f.sp. *tritici*), *P. recondita*	PUCCRT
Septoria leaf blotch, (Septoria tritici blotch)		STB	*Zymoseptoria tritici* (*Septoria tritici*)	SEPTTR
Septoria nodorum blotch		SNB	*Parastagonospora nodorum* (*Septoria nodorum*) (*Phaeosphaeria nodorum*)	LEPTNO
Yellow rust (Stripe rust)		YLR	*Puccinia striiformis* f.sp. *tritici*	PUCCST
Net blotch		NTB	*Pyrenophora teres* (*Drechslera teres*)	PYRNTE
Rhynchosporium (Scald of cereals, leaf blotch of cereals)		RYS	*Rhynchosporium secalis, Rhynchosporium commune*	RHYNSE
Dwarf leaf rust		DLR	*Puccinia hordei*	PUCCHD

We used only data from varieties tested for at least three years to achieve a good representation of the trial conditions and build on a solid database (Mackay et al. 2011). Data included in this study are shown in Table 2. The data set was highly non-orthogonal with respect to variety-year combinations, whereas the variety/location combinations were orthogonal within year and trial series, i.e. all varieties were grown together at all locations within the same year and trial series. The data were checked for recording errors and outliers by calculating standardized residuals based on Eqs. (1) and (2). We excluded observations with standardized residuals greater than + / − 5.0 from further analysis.

**Table 2:** Overview on the data base of German variety trials conducted during 1988–2019

	Total number of				Standard varieties			Average no. per year	
Crops	Observations	Varieties	Locations	Trials	No	First testing year	Average age	Trials	Varieties
Winter wheat	44,253	748	115	1,577	49	1963	7.0	49	74
Winter triticale	10,934	130	90	1,121	34	1987	6.9	35	16
Winter rye hybrid	9,283	121	106	1,281	20	1982	7.2	40	14
Winter rye Population	4,312	37	106	1,281	10	1974	8.4	40	5
Winter barley 2-row	18,201	280	126	1,417	32	1972	6.8	44	29
Winter barley 6-row	17,185	255	119	1,415	36	1975	6.4	44	27
Spring barley	25,210	366	112	1,549	39	1971	7.2	48	38

Stem stability and disease severity was assessed visually at a 1-to-9 scale by crop experts in the field according to the guidelines of the Federal Plant Variety Office (Bundessortenamt 2000). For LDG a score of 1 corresponded to “no lodging, all stems upright”, 3 to “inclination of stems by 30 degrees from upright position or one fourth of the plot shows stronger lodging”, 5 to “inclination of stems by 45 degrees from upright position or one half of the plot shows clustered lodging”, 7 to “inclination of stems by 60 degrees from upright position or total lodging on three quarters of the plot” and 9 to “total lodging”. SBL had to be observed at the lower two third part of the stem before harvest, and EBL buckling at the upper third part before harvest. For both traits score 1 corresponded to “missing”, 3 to “low”, 5 to “medium”, 7 to “strong” and 9 to “very strong” expression. Even values of scores corresponded to intermediate states.

Diseases were scored at two to three different growth stages for each disease following the BBCH-code (Hack et al. 1992). It should be stated clearly that in the VCU trials of this study, plants were not inoculated with diseases, but disease severity was caused by natural field infection. If disease severity was assessed before BBCH 32 (stem elongation, two nodes detectable), it was scored at the whole-plant level. After growth stage BBCH 32, severity was assessed at the two adjacent leaves showing the highest severity. For all plants throughout the trial, severity was rated from the same two leaves. Then, the whole-plot severity was assessed taking into account diseased leaf area and frequency of diseased plants. The transformation of the average percentage of disease-infected leaf area of a plot into a 1 to 9 scale corresponded approximately to a logarithmic transformation according to following scale: score 1 0%, 2 0% – 2%, 3 2% – 5%, 4 5% – 8%, 5 8% – 14%, 6 14% – 22%, 7 22% – 37%, 8 37% – 61%, 9 61% (Bundessortenamt 2000, Sect. 4.1). The recorded score represented the average disease severity of the plot.

**Fig. 2:**
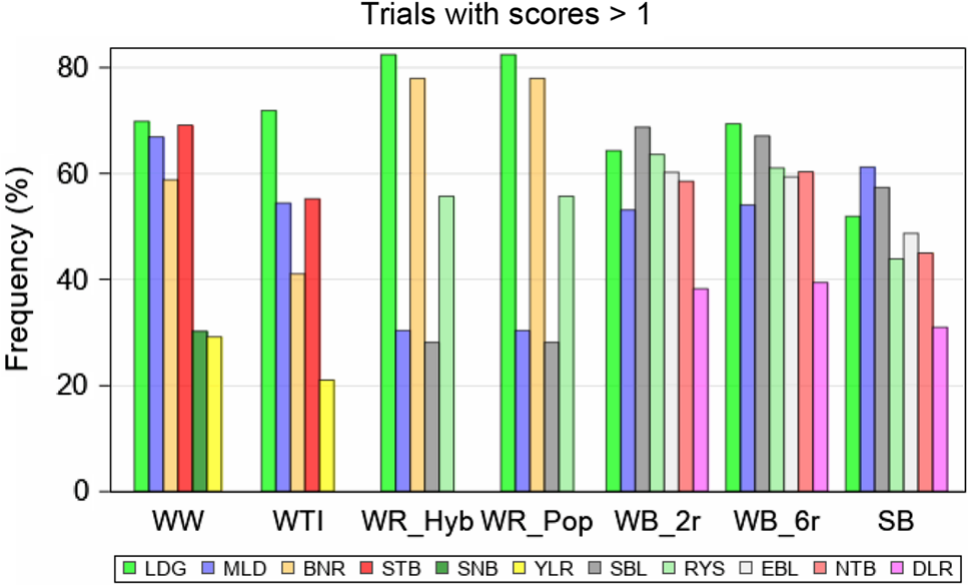
Percentage of trials showing scores > 1 relative to total number of trials. *WW* Winter wheat; *WTI* Winter triticale; *WR* Winter rye, *Hyb* Hybrid, *Pop* Population varieties; *WB* Winter barley, *2-row* two-row, *6-row* six row varieties; *SB* Spring barley; *LDG* Lodging; *SBL* Stem buckling; *EBL* Ear buckling; *MLD* Powdery mildew; *BNR* Brown rust; *STB* Septoria leaf blotch; *RYS* Rhynchosporium; *YLR* Yellow rust; *SNB* Septoria nodorum blotch; *NTB* Net blotch; *DLR* Dwarf leaf rust

Disease severity was scored individually for each replication, intensity level and variety. For the variety registration process, only observations of that growth stage were used, which showed the most clearly visible severity differentiation (i.e. not necessarily the maximum) among varieties. According to Zadoks and Schein (1979, p. 64), this method could be considered as the “critical time method.” Observed disease severity scores at this growth stage were averaged over replications. Only these average values were available for the present study’s analyses. Throughout this paper we used the following terms: (i) “disease severity” to describe each individual variety’s actual visually observed diseased leaf or spikelet area, expressed in the above described “scores”, (ii) “trial disease severity (TSv)” to describe the average disease severity within a trial, calculated as the mean severity score over all varieties in the specific trial, (iii) “variety disease susceptibility (VSc)” to refer to the average disease severity of a variety, calculated as the mean severity score across all diseased trials in which this specific variety was grown. Disease susceptibility of a variety might also be considered as the inverse of its “disease resistance”. A trial was considered as non-diseased with respect to a specific disease, if no disease symptoms for the specific disease were visible for all varieties in this trial, or, if only a few varieties showed a severity score of at most 2 and the others of 1. We further categorized the traits into two groups, (i) the measured traits YLD, EAD, EAY, HGT and EAE as “continuous” traits and (ii) the visually observed traits as “score” traits. For the score traits, the number of trials from which observations were available could be notably smaller than for continuous traits, because scores were only recorded from those trials which were diseased or showed lodging (Fig. 2).

The fertilizer, fungicide and growth regulator rates were recorded in detail for each individual trial and intensity. Nitrogen application rates were accumulated as total kg N ha^−1^. The preceding crops’ residual nitrogen supply was additionally considered in our analysis, based on Table 7 in DUEV (2017), and added to the applied mineral N rate. The nitrogen equivalent of sporadically applied organic fertilizer was considered and added according to the applied mineral N rate. Unfortunately, no data of plant-available mineralized nitrogen in the soil (Nmin) before sowing was available. Fungicide and growth regulator application rates were standardized using the treatment frequency index (TFI) following Roßberg (2006). Many studies used the TFI to assess plant protection intensities in crop production (e.g. Klocke et al. 2020; Strehlow et al. 2020). Here, the TFI described the amount of plant protection products applied to a specific land unit relative to the application amount recommended by the approval authority for each individual plant protection product for the specific crop. A TFI of 1 might derive from the application of a single plant protection product in recommended full dose, but might also derive from the application of two plant protection products, each applied at half the recommended dose. The TFI was often derived separately for fungicides, herbicides, insecticides and growth regulators. Accordingly, we derived separate TFIs for fungicide, growth regulator and herbicide applications in our study. The average rate of applied nitrogen, fungicides, growth regulators and herbicides are shown in Fig. 1.

### Statistical analysis

#### General remarks

Stem stability and diseases were assessed on an ordinal 1-to-9 scale, but we analysed them as being on a quantitative scale. This was standard procedure in the analysis of variety trials and, in fact, decisions for registration of new varieties were based on such analyses (e.g. Zhang et al. 2007). Whereas the scoring data cannot strictly meet the usual assumptions of normality and homogeneity of variance, residual analysis revealed no gross departures (we refer to this later in  section "Statistical limitations of this study", and so we analysed these data as if they were on a quantitative scale.

#### Basic model

For a given observation, we used the standard three-way model with factors genotype, location and year given by Laidig et al. (2008)

1 $$y_{ijk} = \mu + G_{i} + L_{j} + Y_{k} + \left( LY \right)_{jk} + \left( GL \right)_{ij} + \left( {{GY}} \right)_{ik} + \left( {{GLY}} \right)_{ijk} ,$$

where *y*_*ijk*_ was the mean yield of the *i*th genotype in the *j*th location and *k*th year, *μ* was the overall mean, *G*_*i*_ was the main effect of the *i*th genotype, *L*_*j*_ was the main effect of the *j*th location, *Y*_*k*_ was the main effect of the *k*th year, (*LY*)_*jk*_ was the *jk*th location × year interaction effect, (*GL*)_*ij*_ was the *ij*th genotype × location interaction effect, (*GY*)_*ik*_ was the *ik*th genotype × year interaction effect, and (*GLY*)_*ijk*_ was a residual comprising both genotype × location × year interaction and the error of a mean arising from sampling the replications. All effects except *μ*, and *Y*_*k*_ were assumed to be random and independent with constant variance for each effect.

#### Trend for breeding progress based on variety means and gaps

The trend of breeding progress was based on variety means using a quadratic model given by

2 $$\it y_{i} = \mu + \alpha r_{i} + \beta r_{i}^{2} + C_{m\left( i \right)} + V_{i} ,$$

where $y_{i} = \frac{1}{{n_{i} }}\mathop \sum \nolimits_{{{jk}}} y_{{{ijk}}}$ was the mean of genotype *i*, averaged over all $n_{i}$ trials in which the *i*th genotype was present, the covariate *r*_*i*_ was the first trial year of the *i*th genotype. If a variety was in trial for more than 3 years, which was the case for reference varieties, we used only the trials within the first 3 testing years to assess a varieties performance before registration, such that the means of all varieties were based on 3 years. We explain the reasons for using this approach rather than estimating adjusted least square means for varieties in section “Limitations of this study”. For score traits, the frequency of diseased trials, and hence the number of observations for individual variety means, might be different from year to year. We therefore applied a weighted regression analysis with weight $w_{i}\,=\,n_{i}$. There were *m* groups of genotypes with the same first testing year *r*_*i*_, represented by categorical variable *C*_*m(i)*_, where the *i*th genotype had been assigned to the *m*th group. *C*_*m(i)*_ was a random deviation of the *m*th group from the quadratic regression line with variance $\sigma_{C}^{2} ,$ and *V*_*i*_ was a random deviation of the *i*th genotype from group *C*_*m(i)*_ with constant variance $\sigma_{V}^{2}$. The change between 2017 and 1988 was calculated for I2 and I1 by the difference of the prediction for year 2017 and 1988 given by

${\text{Diff}} = E\left( {y_{i}\mid r_{i} = 2017} \right) - E\left( {y_{i}\mid r_{i} = 1988} \right) =\alpha \left( {2017 - 1988} \right) +\beta \left( {2017^{2} - 1988^{2} } \right)$ using Eqs. (1) and (2). It should be noted that 2017 was the last “first testing year” in the data 1988–2019.

Further, gaps between predictions for I2 and I1 (I2–I1) at first trial year 1988 and 2017 were calculated to indicate the difference between intensity levels in 2017 and 1988 (treated with fungicides and growth regulators vs untreated).

#### Trends for ageing effects of varieties

To assess trends of ageing effects for varieties we included all traits. Ageing trends were considered for varieties with more than 10 trial years, which were only reference varieties, so that an ageing effect may be observable. We could not assume that ageing effects at I2 were fully controlled by fungicide and growth regulator application. Therefore, we calculated three different trends: (1) trend for the response at I2, (2) trend for the response at I1 and (3) trend for the difference of the response at both intensities (I2–I1). Further, we assumed that besides the ageing trend, a time trend might be present overlaying the ageing trend such that both trends were confounded. We applied mixed models with linear regression coefficients given by expanding Eq. (1) by a fixed linear regression term as

3 $$ \eta_{ikh} =\mu_{{{ih}}} + \delta_{ih} a_{ik}$$

where $a_{ik} = t_{k } - r_{i}$ was the age of variety *i* at year *k,* and *h* = 1, 2 denoted the intensity, $\mu_{ih}$ was the fixed intercept of the regression slope of variety *i* and intensity *h*, $\delta_{ih}$ was the regression coefficient for ageing effect of variety *i* and intensity *h*. Equation (3) holds, if no additional time trend was present. If we now extended Eq. (3) by an additional time trend for calendar year overlaying the ageing trend, then we obtained

4 $$ \eta_{ikh} =\mu_{ih} + \gamma_{h} t_{k } + \delta_{ih} a_{ik} ,$$

where $\gamma_{h}$ was a fixed regression coefficient for time trend for intensity *h* and $t_{k }$ was the continuous covariate for the calendar year.

We further assumed that $\delta_{ih} =\delta_{h} + V_{ih}$, where $\delta_{h}$ was a common regression coefficient for ageing and *V*_*ih*_ was a random deviation from the common regression coefficient of variety *i* and intensity *h*, which was considered to be independently distributed with variance $\sigma_{Vh}^{2}$ (Piepho and Ogutu 2002). Then the fixed linear regression term of Eq. (4) reduced to

5 $$ \eta_{ikh} = \mu_{ih} + \gamma_{h} t_{k } + \delta_{h} a_{ik}$$

When $\gamma_{h} r_{i } - \gamma_{h} r_{i }$ was added to Eq. (5) and rearranged, then we got

6 $$ \eta_{ikh} = \tilde{\mu }_{ih} + \tilde{\delta }_{ih} a_{ik} ,$$

where

7 $$\tilde{\mu }_{ih} ={\mu } _{ih} + \gamma_{h} r_{i }$$

was the apparent intercept of variety *i* and

8 $$ \tilde{\delta }_{ih} = (\delta_{h} + \gamma_{h} )a_{ik}$$

was the apparent ageing effect taking into account a time trend.

We considered not only the ageing effect of both intensities separately, but further also the difference of ageing trend I2–I1, given by

9 $$ \eta_{ik2} - \eta_{ik1} = \left( {\tilde{\mu }_{i2} - \tilde{\mu }_{i1} } \right) + (\tilde{\delta }_{i2} - \tilde{\delta }_{i1} )a_{ik}$$

If we denoted $\left( {\tilde{\mu }_{i2} - \tilde{\mu }_{i1} } \right)$ by $\Delta \tilde{\mu }_{i}$ and $\left( {\tilde{\delta }_{i2} - \tilde{\delta }_{i1} } \right)$ by $\tilde{\delta }_{i}$, then the fixed regression terms for ageing trend between I2 and I1 was given by

10 $$ \eta_{ik2} - \eta_{ik1} = \Delta \tilde{\mu }_{i} + \tilde{\delta }_{i} a_{ik}$$

From Eq. (7), we saw that the apparent intercept was a fixed effect, given as the sum of a constant and the product of the regression coefficient of the time trend and the variety’s first trial year. We modelled fixed intercepts for varieties to eliminate trends due to genetic progress. The apparent ageing trend was composed of the ageing trend and the time trend. Unfortunately, the intercept $\mu_{ih}$ and the ageing trend $\delta_{h}$ were not estimable, because they were linearly dependent. This is an inherent property of long-term variety trial data (Piepho et al. 2014).

#### Genotypic and environmental variation

We estimated variance components to quantify genotypic, environmental and interaction of genotype environmental variation of crops and their traits using Eq. (1). Estimates of long-term variance components might be biased if time trends are present in random effects. Consequently, trends in traits might result in larger components for genotypic variance. Therefore, we extended Eq. (1) by regression terms to model time trends. To remove possible bias due to trends, we assumed a nonlinear trend in the genotypic effects given by

11 $$ G_{i} =\beta_{1} r_{i} + \beta_{2} r_{i}^{2} + H_{i} ,$$

where $r_{i}$ was the first testing year of genotype *i* and $H_{i}$ was a random deviation from the genotypic trend. Further, in the year effect, we assumed a linear time trend by

12 $$ Y_{k} =\gamma t_{k} + Z_{k} ,$$

where $t_{k}$ was the testing year *k*, and $Z_{k}$ was a random deviation from the linear non-genetic trend.

Then we estimated the variance components of the random effects of the extended model by assuming that the effects were independent and identically normally distributed.

## Results

### Treatment intensities

For a valid classification and better interpretation of the following results, the trait-specific treatment intensities are graphically provided in Fig. 1. From 2005 onwards, nitrogen application rates were identical in both intensities for all crops. Accordingly, N rates increased in I1 and were reduced in I2 by about 10 to 50 kg ha^−1^, respectively, depending on the crop (Fig. 1). Furthermore, nitrogen application levels were lowest in SB and highest in WW. In all crops, except WR, a rather linear increase in nitrogen application rates can be observed. In WR application rates did not increase after 2005. Fungicide applications showed a decreasing trend until about 2000 followed by increases especially after 2011, however, at different levels in the different crops. Here again, in SB the TFI was only about half as high compared to WW. The TFIs of growth regulators increased rather linearly and approximately doubled over the investigated time period, with WR having the highest TFI of all crops and being the only crop where I1 received growth regulator treatment. Only in SB growth regulator TFI increased rather marginally. Herbicide application increased sharply in the first few years and then showed only a slightly increasing trend without much differentiation between crops.

### Trend for breeding progress based on variety means and gaps

**Fig. 3:**
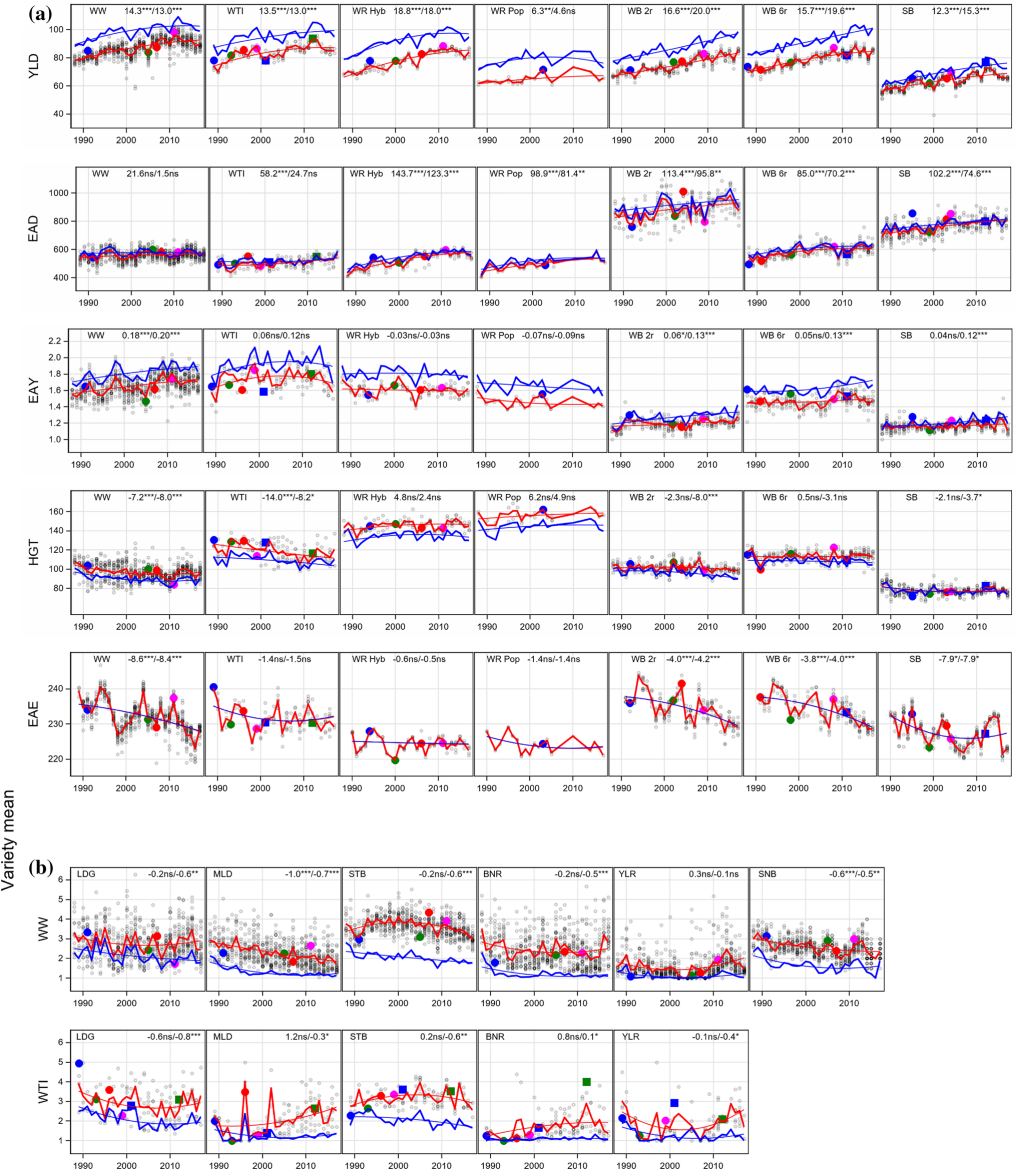

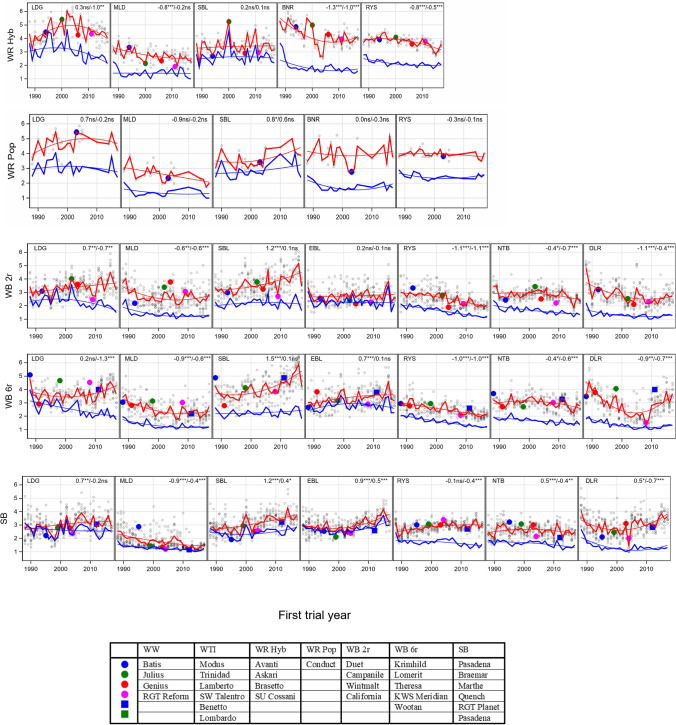
Breeding progress in German cereal crops for (**a**) continuous and (**b**) score traits. Variety means in intensity (I1) (grey circles) are plotted against first trial year 1988–2017. Filled colored circles and squares highlight landmark varieties. For SB variety means of EAE are shifted by plus 150 days. The red line shows year means in I1 and blue in intensity 2 (I2). Quadratic regression lines are indicated for I1(red) and I2(blue). As insets at the top right are given: absolute changes over 1988–2017, where the left figure represents change in I1, and the right one in I2. *WW* Winter wheat; *WTI* Winter triticale; *WR* Winter rye, *Hyb* Hybrid *Pop* Population varieties; *WB* Winter barley, *2r* two-row, *6r* six row varieties; *SB* Spring barley; *LDG* Lodging; *SBL* Stem buckling; *EBL* Ear buckling; *MLD* Powdery mildew; *BNR* Brown rust; *STB* Septoria leaf blotch; *RYS* Rhynchosporium; *YLR* Yellow rust; *SNB* Septoria nodorum blotch; *NTB* Net blotch; *DLR* Dwarf leaf rust; *YLD* Yield (dt ha^−1^); *EAD* Number of ears per m^2^; *EAY* Single ear yield (g); *HGT* Plant height (cm); *EAE* Days from sowing to ear emergence; *I1* Intensity 1; *I2* Intensity 2; *ns* non-significant; *Significant at 5% level; **Significant at 1% level; ***Significant at 0.1% level

The number of observations (trials) for a specific variety mean varied considerably among stem stability and diseases, because not at each trial an incidence was observed. As can be seen from Fig. 2, the frequency of occurrence was very different between traits. LDG occurred very frequently, whereas some diseases occurred only less than half as frequent, e.g. YLR. However, varieties with the same first trial year had always identical numbers of observations. As mentioned earlier, we calculated trial means only on basis of a variety’s first three trial years. Figure 3 illustrates the breeding progress, where insets at the top right of the individual subplots indicate the absolute changes during 1988–2017 in I1 (left number) and I2 (right number). Landmark varieties were displayed to provide orientation across different traits, as they are popular varieties with considerable acreage in practical farming during a longer period with well-known susceptibility characteristics. Additionally, Table 3 shows trait levels of first trial year 1988 and 2017 in I1 and I2 and the difference between levels in 1988 and 2017 in I2 and I1. Further, gaps between levels I2 and I1 (I2–I1) in 1988 and 2017 and the difference between gaps 2017–1988 were displayed. Most of the trends were linear or showed a slight deviation from linearity, while the shape of nonlinearity differed between traits (data not shown). Nonlinear shapes were diminishing increasing (e.g. in WW and WR Hyb for YLD), concave (e.g. in WB and SB for DLR) or convex (e.g. in WR Hyb for LDG, in WW for STB).

**Table 3:**
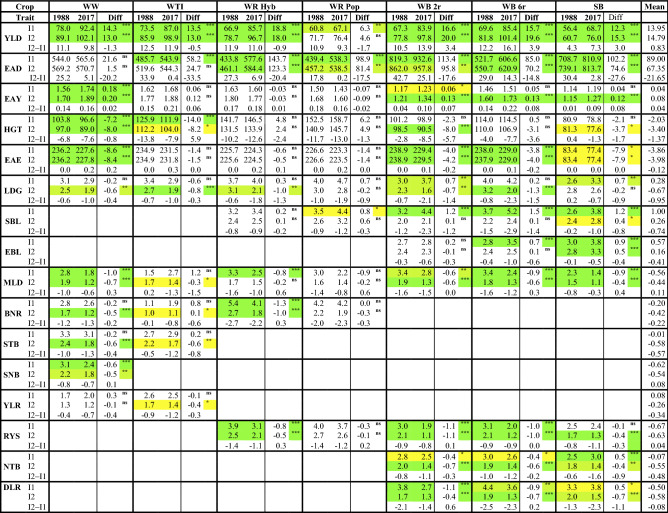
Breeding progress in German cereal crops

Trends of variety means showing trait levels of first trial year 1988 and 2017 in I1 and I2 and the difference between levels 1988 and 2017. Further, gaps between levels I2−I1 at 1988 and 2017 and the difference between gaps 2017–1988. Green colored cells represent significance at 0.1%, green-yellow at 1% and yellow at 5% levels. For continuous traits (except for HGT) positive changes indicate an improvement over time, but for score traits negative changes indicate an improvement

*WW* Winter wheat; *WTI* Winter triticale; *WR* Winter rye; *Hyb* Hybrid, *Pop* Population varieties; *WB* Winter barley, *2r* two-row, *6r* six row varieties; *SB* Spring barley; *YLD* Yield (dt ha^−^); *EAD* Number of ears per m^2^; *EAY* Single ear yield (g); *HGT* Plant height (cm); *EAE* Days from sowing to ear emergence; *LDG* Lodging; *SBL* Stem buckling; *EBL* Ear buckling; *MLD* Powdery mildew; *BNR* Brown rust; *STB* Septoria leaf blotch; *RYS* Rhynchosporium; *YLR* Yellow rust; *SNB* Septoria nodorum blotch; *NTB* Net blotch; *DLR* Dwarf leaf rust; *I1* intensity 1; *I2* intensity 2

^ns^Non-significant; *Significant at 5% level; **Significant at 1% level; ***Significant at 0.1% level

In the following, we first considered the continuous traits YLD, EAD, EAY, HGT and EAE, which were observed in all crops and trials. Figure 3a shows large **YLD** (acronym of trait are boldfaced at start of passage following with detailed results) increases for all crops in I1 and I2, except for WR Pop. YLD I2 increased in the range of 4.6 dt ha^−1^ (WR Pop) and 20.0 dt ha^−1^ (WB 2r) and YLD in I1 between 6.3 dt ha^−1^ (WR Pop) and 18.6 dt ha^−1^ (WR Hyb). YLD gaps (I2-I1) in 1988 varied in the range of 4.3 dt ha^−1^ (SB) and 12.5 dt ha^−1^ (WTI). Comparison of YLD gaps 1988 with 2017 revealed two groups of crops (Table 3). In WW (− 1.3 dt ha^−1^), WTI (− 0.5 dt ha^−1^), WR Hyb (− 0.9 dt ha^−1^) and WR Pop (− 1.7 dt ha^−1^) gaps decreased, whereas in WB 2r (3.4 dt ha^−1^), WB 6r (3.9 dt ha^−1^) and SB (3.0 dt ha^−1^) gaps increased. **EAD** varied over a wide range between crops with lowest values in WR (about 440–580 ears m^−2^) and highest in WB 2r (about 820–960 ears m^−2^). EAD increased for all cereals in I1 and I2, especially strongly in WR Hyb (143.7 ears m^−2^ in I1, 123.3 ears m^−2^ for I2).

Contrary to WR Hyb, EAD in WR Pop increased by only 98.9 ears m^−2^ in I1 and 81.4 ears m^−2^ in I2. The increase in I1 was generally notably higher than in I2, which resulted in a reduction in gaps 2017 between − 14.8 ears m^−2^ (WB 6r) and − 33.5 ears m^−2^ (WTI). **EAY** increased in all crops in I1 and I2, but not in WR, where EAY decreased. Figure 3a further shows increasing gaps between 1988 and 2017 in the range of 0.01 g (WTI) and 0.08 g (WB 6r and SB) per ear. **HGT** I1 was considerably reduced in WTI (− 14.0 cm) and WW (− 7.2 cm), whereas in WR and WB 6r HGT I1 increased slightly. HGT in I2 changed more than in I1 resulting in narrowing HGT gaps. The trend pattern for **EAE** was nearly the same in I2 and I1, therefore only the trends in I1 were plotted in Fig. 3a. In WW the period between sowing and ear emergence was reduced by 8.6 days followed by SB with 7.9 days, while not much change was found in WR and WTI.

Next, we looked at traits for stem stability LDG, SBL and EBL shown in Fig. 3b. When considering score traits, we should note that signs of changes and gaps need a reversed interpretation. For score traits, a positive change indicated increased susceptibility, i.e. a negative progress. Furthermore, negative gaps indicated a widening of gaps over time, i.e. that susceptibility in I1 increased stronger than in I2. **LDG** in I1 improved in WW (− 0.2) and WTI (− 0.6), while for other crops susceptibility in I1 increased between 0.2 (WB 6r) and 0.7 (WB 2r and SB). When comparing differences of gaps between 2017 and 1988, we found a widening among crops ranging from 0.3 in WTI to 1.5 in WB 6r, which means that LDG increased up to 1.5 scores during 1988 and 2017, and stronger in I1 than in I2. Susceptibility of varieties for **SBL** and **EBL** increased in WR, WB and SB in I1 and I2 resulting in increasing gaps.

Finally, we considered the diseases (Fig. 3b, Table 3). Trends in I1 represented breeding progress for disease resistance when no fungicides and growth regulators were applied, but the trend for I2 represented breeding progress under fungicide and growth regulator treatment. **MLD** was observed in all crops, showing a decreasing trend in I1 and I2, except in WTI, where the susceptibility level increased by 1.2 in I1, but decreased by − 0.3 in I2. Susceptibility across crops in I1 decreased in the range of − 1.0 (WW) and − 0.6 (WB 2r), which showed that susceptibility levels for MLD were considerably higher in WR and WB compared to WW, WTI and SB. In all crops, the gaps decreased, but it widened in WTI by −1.5 scores, which means that the difference in susceptibility between I2 and I1 was 1.5 scores larger in 2017 compared to 1988. We found a strong decrease in susceptibility for **BNR** in WR Hyb (− 1.3 for I1, − 1.0 for I2), but in WTI susceptibility increased (0.8 in I1, 0.1 in I2). However, gaps decreased in WW, WR, but increased in WR Hyb by 0.3 scores. Notably, WR Hyb showed the highest BNR susceptibility level and WTI the lowest. **STB** susceptibility decreased only slightly in I1 in WW and WTI; however, in I2, the decrease was larger in both crops. As shown in Table 3, gaps in WW and WTI widened. **SNB** was only observed in WW showing a significant decrease of − 0.6 in I1 and − 0.5 in I2. **YLR** did not change in I1 in WW and WTI, and decreased slightly in WTI I2 (− 0.4), but gaps increased in WW (0.4) and WTI (0.3).

Breeding toward lower susceptibility for **RYS** I1 was successful in all crops, especially in WB 2r (− 1.1) and WB 6r (− 1.0). **NTB** susceptibility decreased by − 0.4 in WB 2r and 6r for I2, and by − 0.7 in WB 2r and − 0.6 in WB 6r in I2. While susceptibility also decreased by − 0.4 in SB I2, it increased by 0.5 in SB I1, so that the gap in SB increased by 0.9. **DLR** I1susceptibility declined by − 1.1 in WB 2r and − 0.9 in WB 6r, but increased by 0.5 in SB I1. In WB 2r, WB 6r and SB I2, DLR susceptibility reduced by − 0.4, − 0.7 and − 0.7 in I2, respectively. In SB the gap remarkably increased by − 1.1, while in WB a considerable decrease was found. As Fig. 3b indicates, DLR susceptibility noticeably dropped until 2010 and 2015 in WB and SB, respectively, and then increased strongly.

### Trends for ageing effects of varieties

Table 4 shows the results of the estimated ageing effects of reference varieties over 10 years in I1, I2 and the absolute difference I2−I1. Further details of the regression coefficients for ageing effects are shown in Supplementary Material SM3, Table S1. While the number of varieties differed strongly between crops ranging from 7 in WR Pop to 49 in WW, differences in average age were much smaller ranging from 7.2 years in WW to 9.9 years in WR Pop. Besides ageing effects in I2−I1  *d*_21_), we showed the effect in I2 (*d*_2_) and I1(*d*_1_) separately, since they differed greatly in terms of sign and magnitude. Knowing ageing effects in I2 and I1 was helpful to interpret the ageing effects *d*_21_. In most cases, the difference between the change estimated in I1 and I2 was very similar to the differences *d*_21_. However, standard errors for individual regression coefficients of *d*_1_ and *d*_2_ were larger than for *d*_21_, because random effects for the differences I2−I1 had smaller variances than the corresponding random effect of I1 and I2, separately (data not shown). In the following we focused mainly on the ageing effect *d*_21_.

**Table 4:**
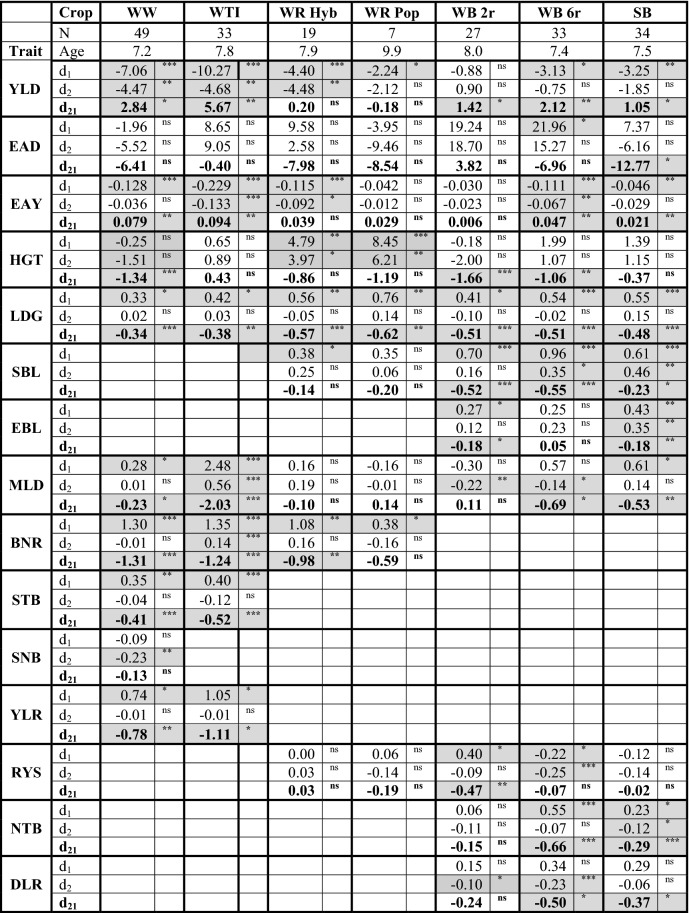
Effect of variety ageing for reference varieties over 10 years by using Eqs. (6) and (8)

*d*_1_ represents the change in I1, *d*_2_ in I2, and *d*_21_ in difference I2–I1. *Age* is the average maximum age of all reference varieties, *N* the number of reference varieties. Grey marked cells show significant age trends. For continuous traits, a positive sign of *d*_21_ indicates a stronger decrease of age trend for I1 than for I2. For score traits, a negative sign of *d*_21_ indicates a stronger increase of susceptibility for I1 than I2

*WW* Winter wheat, *WTI* Winter triticale; *WR* Winter rye, *Hyb* Hybrid, *Pop* Population varieties; *WB* Winter barley, *2r* two-row, *6r* six row varieties; *SB* Spring barley; *YLD* Yield (dt ha^−1^); *EAD* Number of ears per m^2^; *EAY* Single ear yield (g); *HGT* Plant height (cm); *EAE* Days from sowing to ear emergence; *LDG* Lodging; *SBL* Stem buckling; *EBL* Ear buckling; *MLD* Powdery mildew; *BNR* Brown rust; *STB* Septoria leaf blotch; *RYS* Rhynchosporium; *YLR* Yellow rust; *SNB* Septoria nodorum blotch; *NTB* Net blotch; *DLR* Dwarf leaf rust

^ns^Non-significant; *Significant at 5% level; **Significant at 1% level; ***Significant at 0.1% level

We first looked at continuous traits YLD, EAD, EAY and HGT. We should note that for continuous traits positive ageing effects for *d*_21_ mean that the decrease *d*_1_ was larger than for *d*_2_. For **YLD**, we found significant negative ageing effects *d*_21_ in all crops except in WR. In WR negative ageing effects of similar strength were found for *d*_1_ and *d*_2_, which led to an ageing effect *d*_21_ of nearly zero. WTI and WW showed the largest ageing effects of *d*_21_ = 5.67 dt ha^−1^ and *d*_21_ = 2.84 dt ha^−1^, respectively. The individual changes for *d*_2_ and *d*_1_ indicated that also in the treated intensity I2 considerable decreases appeared in the different crops, but generally smaller than in I1. This gave evidence that the ageing effect *d*_21_ differed from the ageing effect *d*_1_. For **EAD** all ageing effect *d*_21_ were non-significant. For the yield component **EAY**, a similar ageing pattern as for YLD was apparent. **HGT** showed significant negative trends in WW (*d*_21_ =  − 1.34 cm), WB 2r (*d*_21_ =  − 1.66 cm) and WB 6r (*d*_21_ =  − 1.06 cm). When comparing the ageing effects in I2 and I1 for these crops, it became apparent, that HGT reduction in I2 was larger than in I1 (WW, WB 2r) or in the case of WB 6r, HGT increase in I2 (*d*_2_ = 1.07 cm) was smaller than in I1 (*d*_1_ = 1.99 cm). This means that over 10 years HGT reduced on average by *d*_21_ =  − 1.34 cm in WW, *d*_21_ = −1.66 cm in WR Hyb and *d*_21_ = − 1.06 cm for WR Pop.

Next, we considered the score traits LDG, SBL and EBL, which represented the physical stem stability at the stage of ripeness. Please note that for score traits, negative ageing effects in I2−I1 (*d*_21_) meant that increase of susceptibility in I1 (*d*_1_) was higher than for I2 (*d*_2_). A positive value for ageing effect *d*_1_ and *d*_2_ indicated increasing susceptibility, while a negative value for *d*_21_ indicated increasing susceptibility. For nearly all crops, we observed an increased susceptibility towards LDG. Moreover, WB and SB showed increasing susceptibility for SBL and EBL.

Among diseases, ageing effects for **MLD** were present in all crops, except in WR and WB 2r. A notably large decrease was revealed for WTI (*d*_21_ =  − 2.03). Furthermore, WW and WTI were subject to large **BNR** ageing effects of *d*_21_ =  − 1.31 and *d*_21_ =  − 1.24, respectively. In addition, the susceptibility for **STB** and **YLR** increased with age in both crops, while susceptibility for **RYS** was rather stable except for WB 2r (*d*_21_ =  − 0.47). Ageing effects for **NTB** and **DLR** were identified in WB 6r (*d*_21_ =  − 0.66, *d*_21_ =  − 0.29) and SB (*d*_21_ =  − 0.50, *d*_21_ =  − 0.37), respectively.

### Genotypic and environmental variation

**Fig. 4:**
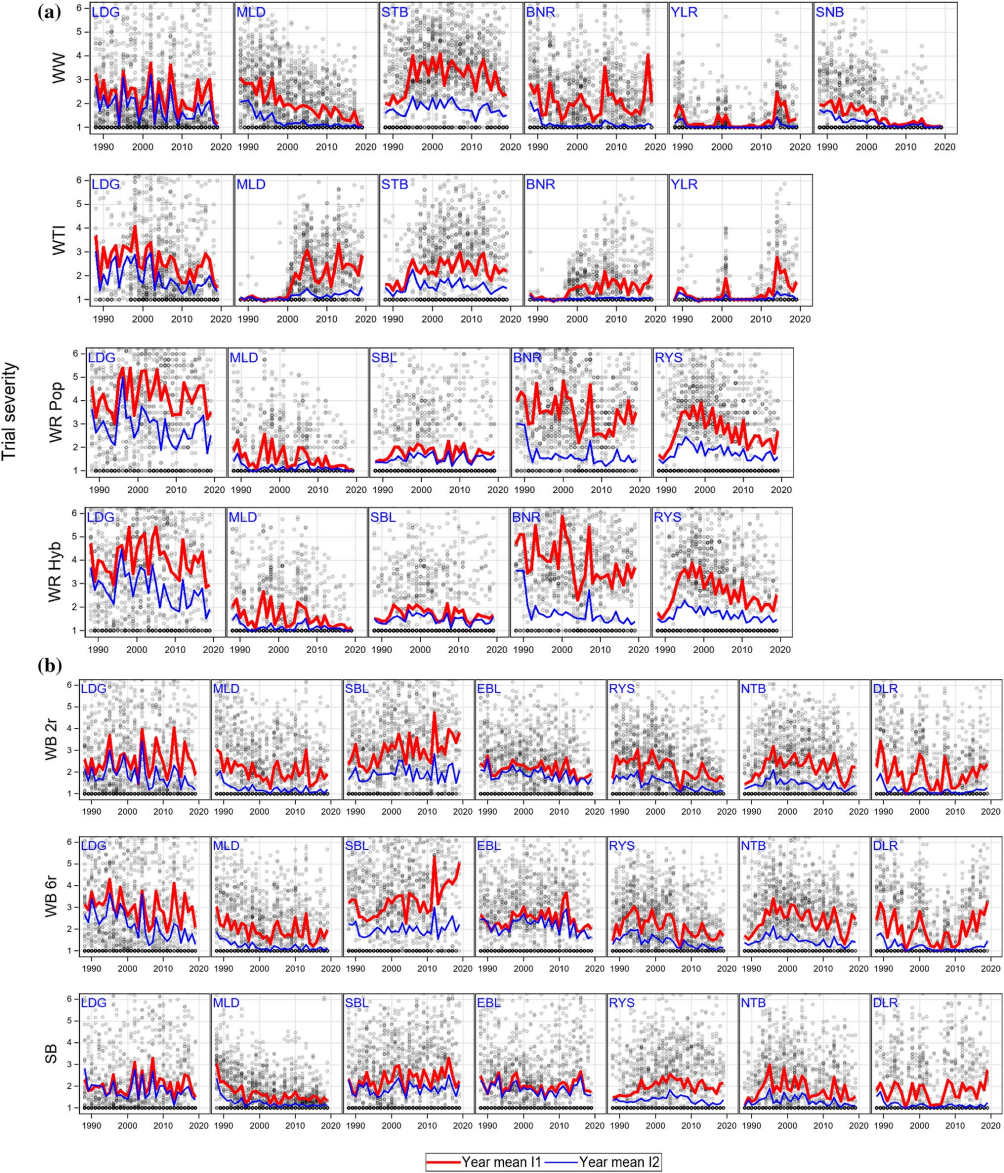
Trial severity (TSv) 1988—2019 for stem stability and diseases across all varieties within a trial. Grey circles represent the within trial average, the red line the year average in intensity 1, the blue line the year average in intensity 2. Trials with scores 1 are included. *WW* Winter wheat; *WTI* Winter triticale; *WR* Winter rye, *Hyb* Hybrid *Pop* Population varieties; *WB* Winter barley, *2r* two-row, *6r* six row varieties; *SB* Spring barley; *LDG* Lodging; *SBL* Stem buckling; *EBL* Ear buckling; *MLD* Powdery mildew; *BNR* Brown rust; *STB* Septoria leaf blotch; *RYS* Rhynchosporium; *YLR* Yellow rust; *SNB* Septoria nodorum blotch; *NTB* Net blotch; *DLR* Dwarf leaf rust

Figure 4 shows variability of TSv scores I1 (grey circles) for crops and traits across years, where the red and blue line represent the year means in I1 and I2, respectively. Non-diseased trials are represented by circles along the 1-score line. All plots show a very large within-year variation of TSv compared to the smaller between-year variation. The line pattern in I1 was very similar for the same diseases and crops, e.g. STB in WW and WTI, NTB, and DLR in WB and SB. The simultaneous epidemic occurrence for YLR in WW and WTI was highlighted. The gradual adaptation of MLD and BNR to WTI from 1995 and 2000 on, respectively, is clearly visible in Fig. 4.

**Fig. 5:**
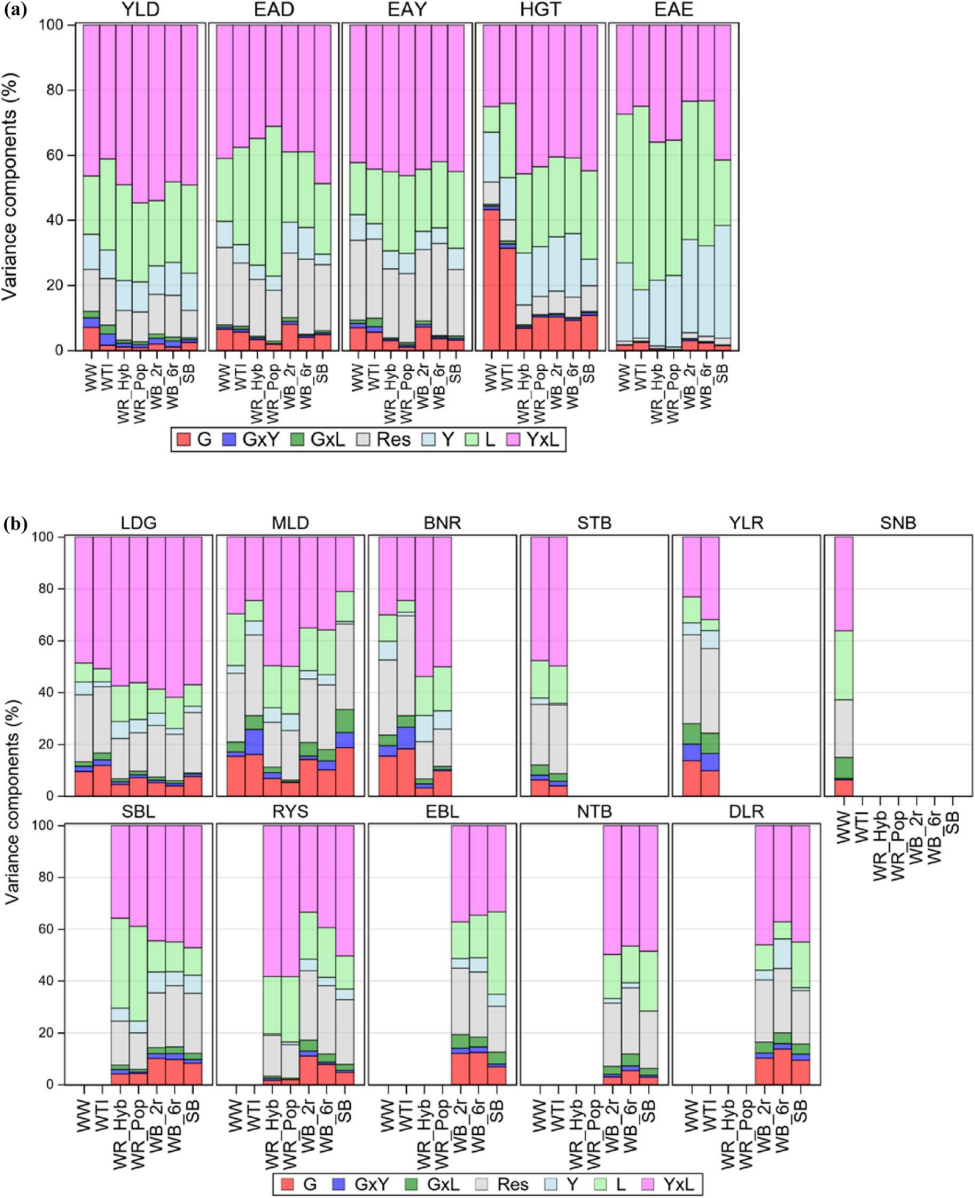
Variance components for (**a**) continuous and (**b**) score traits in intensity 1 as percent of total sum of components using basic model given by Eq. (1) assuming a nonlinear trend in the genotype effect *G*_*i*_ (Eq. (11)) and linear trend in the year effect *Y*_*k*_ (Eq. (12)). *G* Genotype; *GxY*, *GxL*: Genotype by year, location; *Res* Residual;* Y* Year; *L* Location; *YxL*: Year by location; *WW* Winter wheat; *WTI* Winter triticale; *WR* Winter rye, *Hyb* Hybrid, *Pop* Population varieties; *WB* Winter barley, *2r* two-row, *6r* six-row varieties; *SB* Spring barley; *YLD* Grain yield; *EAR* Ear density; *EAY* Ear yield; *HGT* Plant height; *EAE* Days to ear emergence; *LDG* Lodging; *SBL* Stem buckling; *EBL* Ear buckling; *MLD* Powdery mildew; *BNR* Brown rust; *STB* Septoria leaf blotch; *RYS* Rhynchosporium; *YLR* Yellow rust; *SNB* Septoria nodorum blotch; *NTB* Net blotch; *DLR* Dwarf leaf rust

Variance components for continuous and score traits were considered separately and displayed in Fig. 5a, b, respectively. The detailed percentage numbers are listed in Supplementary Material SM4, Table S2. To get an overview on the variation pattern, we averaged variance components across crops and traits for both groups of traits. We found notably different patterns of variation between the two groups of traits. The environmental variation (*Y* + *L* + *YL*) accounted for about 80% on average for continuous and about 60% for score traits, while the residual variance (*RES*) for score traits was about twice as large (23%) as for continuous traits (12%). The influence of years for continuous compared to score traits was three times higher (12% vs. 4%). We found about the same relation between both groups of traits for the genotype-environment (*GY* + *GL*) vs. environmental variation of 5% vs. 2%, respectively. The genotypic variation was low and accounted for only 6% for continuous and 9% for score traits.

We first considered the variation pattern for continuous traits (Fig. 5a). The genotypic variation was very small for EAE and YLD, but large for HGT and moderate for EAD and EAY. For HGT, WW and WTI genotypes showed considerably higher variation than the other crops. In all crops, the component for location (*L*) was higher than that for years (*Y*), except for HGT. The highest variation for HGT was found in WW genotypes. For all crops and traits, the share of *GY* and *GL* variation was nearly negligible. The residual variation for EAE and HGT was considerably smaller than for other continuous traits.

Finally, we looked at score traits (Fig. 5b). We found the largest genotypic variation for MLD, BNR and YLR, especially in WW, WTI and SB, but only a rather low variation for STB, SNB and NTB. In all crops, the highest average variation could be attributed to the *YxL* interaction followed by the residual error. Especially in WTI, the residual error was very large (e.g. for MLD, BNR). The variation of the year effect (*Y*) was much smaller than of the location effect (*L*). The same relation was evident for the interaction of *GY* and *GL*, except for YLR. For YLR larger components were estimated than for any other disease, which were 6% and 7% (*GY*) and 8% and 8% (*GL*) in WW and WTI, respectively.

## Discussion

The primary focus of this study was to quantify trends of breeding progress in cereal crops towards more resistant varieties and to compare the achievements between crops. Yield, yield-related traits and disease traits interact in a complex agro-biological system. They are important traits for breeders to enhance varieties for improved performance and better resilience towards biotic and abiotic stress. Furthermore, they are essential criteria in official variety trials for assessing VCU.

### Trend for breeding progress based on variety means and gaps

We found a notable increase in YLD in all crops in I1 and I2, especially in WB 2r and 6r. The increase in YLD I2 in WW and SB was lower than in WR Hyb and WB, which may be attributed to the fact that besides YLD, considerable progress in baking and malting quality in WW and SB has been achieved (Laidig et al. 2017a, b), hampering part of progress in yield potential in these two crops. Further, nitrogen level in WB and SB increased during 1988–2019, while in WW and WTI it was rather constant (Fig. 1). Comparing YLD gaps (I2−I1) in 1988 and 2017, they were only reduced in WW, WTI and WR, but not in WB and SB (Table 3). One possible reason might be the difference in the development of nitrogen application rates in I2 versus I1 in the different crops as shown in Fig. 1. The differences before 2005 were smallest in WB and SB, while after 2005 only the nitrogen levels in WB and SB continuously increased, but in the other crops were rather constant.

In all crops, increase for EAD was larger in I1 than in I2. As Fig. 3 and Table 3 show, the gaps decreased from 1988 to 2017. This observation may be mainly due to the different nitrogen application rates before 2005. Lower N rates in the early years limited EAD in I1, while this limitation diminished in later years. However, higher nitrogen rates also might have led to higher susceptibility for specific fungal diseases (see, e.g. Veresoglou et al. 2013), which then led to a lower increase for EAY in I1 versus I2 over time.

**HGT** I1 was considerably reduced in WW and WTI. However, in WR Hyb and Pop, even a slight increase was found. In WB and SB, nearly no change in HGT is shown in Fig. 3, which might be due to the fact that no major dwarfing genes were available (Berry et al. 2004). Overall, HGT reduction in I2 was stronger due to the application of growth regulators (Froment and MacDonald 1997), e.g. in WB 2r, HGT was reduced by − 8.0 cm for I2 versus − 2.3 cm in I1. It is remarkable that in WR HGT increased despite increased growth regulator application in I2 and, but at a lower rate, in I1. Photosynthesis is the primary determinant of crop yield (Simkin et al. 2019). In rye, the culm serves as increasingly important reserve storage under stress conditions and main photosynthesis organ (Nalborczyk et al. 1981) with its contribution to overall photosynthesis being higher than in all other cereals (Takeda and Udagawa 1976). Hence, continuous selection for grain yield and stress tolerance may be considered as the main driver for the increase in plant height in both WR variety types. This explanation has a genetic reason but increasing trends of ageing effects for HGT in I1 and I2 (Table 4) suggest that besides a genetic reason also non-genetic effects may be involved leading to increasing trends for HGT.

Berry et al. (2015) evaluated the effect of breeding on HGT in WW during 1970–2013 in UK variety trials. They found a gradual HGT reduction until 1990 and then no significant trend between 1990 and 2013. They argued that breeders probably did not find an advantage in further reducing HGT to increase lodging resistance, because shorter plants would counteract in efforts towards further raising yield. We found a similar trend pattern for WW as depicted in Fig. 3, where a strong decrease until 1991 and then nearly no reduction was visible. Berry et al. (2004) stated “that the minimum height that is compatible with high yield is being approached in some cereals. Therefore, breeders must exploit the large amount of genetic variation in the strength of the stem and anchorage system to continue producing lodging-resistant varieties.” Nevertheless, in WR, the tallest cereal investigated by far in this study, the gibberellin-sensitive dwarfing gene *Ddw1* is currently under research to genetically reduce plant height and improve lodging resistance in future hybrid rye varieties (Rye-sus.eu 2021). The relation between grain yield and HGT was quantified in a study by Casebow et al. (2016). They found a quadratic relationship between grain yield and crop height manipulated by the reduced-height genes with highest yields around 80 cm. They further found an inverse quadratic relationship for nitrogen concentrations in grain at a minimum by about 80 cm.

In all crops, a trend towards shortening of **EAE** became obvious, i.e. earlier ear emergence. Our results were supported by a study of Voss-Fels et al. (2019) including 191 winter wheat varieties released during the last 50 years, mainly in Germany, and grown under contrasting agrochemical inputs over two growing seasons. Their results showed an increase in days from EAE to 1 July under all input levels. This result suggested that besides climate change also genetic effects may have increased days of EAE to 1 July. Bönecke et al. (2020) also reported a reduced number of days until ear emergence in German wheat since the middle of last century. However, in their study, the period did not shorten when expressed in thermal time, which indicates intermingled effects of genetic and climatic change regarding EAE over time.

**LDG** occurred in all crops showing an increasing trend towards higher LDG in I1, whereas in I2 the trend was more or less decreasing. LDG is a complicated phenomenon that is influenced by many factors including plant height, stem strength, wind, rain, topography, soil type, previous crop, husbandry and diseases that attack the basal shoots already during early vegetative growth (Berry et al. 2004). The highest LDG level was found for WR Hyb and Pop (Table 3), which corresponds closely to increased HGT despite increased application of growth regulators in WR. Further, a strong increase of EAD is likely another factor for increased lodging as reported by Matsuyama and Ookawa (2020) in WW varieties. They found that decreasing seed rates increased stem stability and resistance against lodging. From this, one can conclude that increased seed rates and EAD increase the susceptibility for LDG. Additionally, the tendency towards more lodging in I1 in cereals might be due to the increased nitrogen application in I1 (Fig. 1) and thus higher EAY and/or increasing EAD as was observed in WR and WB. The decreasing trend for LDG in I2 could be attributed to the higher application intensity and efficacy of growth regulators (Fig. 1). Overall, progress in lodging tolerance in WW was reported by Berry et al. (2004), Berry et al. (2015) and Zhang et al. (2020) mainly in consequence of reduced plant height and improved application of growth regulators. On the other side, higher nitrogen application rates increasing yield potential have raised the risk for lodging, which might also explain the increasing trend for LDG in other cereals.

A serious increase in **SBL** occurred in WB and SB in I1, but not in I2, which might be attributed to the stem shortening effect. **EBL** increased in WB 6r and SB in I1 and I2 indicating that growth regulator treatment had no reducing effect for this trait. Generally, Fig. 3 shows that stem stability decreased in the untreated trials.

Before discussing the progress of individual diseases towards higher resistance in the different cereals, we should briefly highlight the special situation in WTI. This cereal resulted from the hybridization of wheat and rye and has only been increasingly cultivated in Germany since the 1990s (Oettler 2005). Originally, WTI was completely resistant to the biotrophic pathogens MLD, BNR and YLR because of the absence of adapted pathogen populations. Therefore, no resistance breeding efforts took place. In the official trials, BNR occurred firstly in 1988, YLR in 1989, and MLD in 2001, but YLR and BNR occurred only sporadically on highly susceptible genotypes or at single locations (Fig. 4). A regular appearance or even devastating epidemics were observed for BNR since 1998, for MLD already with the first appearance in 2001, and for YLR in 2014 with the emergence of the Warrior race (see Fig. 4), and these years were also the start of natural resistance selection in the nurseries. From the beginning, there were some varieties protected by wheat genes (*Pm, Lr, Yr,* Audenaert et al. 2014) until the corresponding virulences spread in the newly emerged triticale pathogen populations as a result of the increasing acreage of WTI. At the beginning of this study (1988) WTI was grown in Germany on 20,000 ha, compared to 358,200 ha at the end (2019). In addition, WTI varieties might also contain specific resistance genes from WR. Before the start of resistance selection, resistances in WTI were chance events. Varieties without such resistance genes were susceptible to a much higher degree than WW varieties because they were not protected by quantitative resistances as these were accumulated in modern WW varieties over many years of breeding (Miedaner and Flath 2007; Serfling et al. 2017). The breeding progress for the resistances to biotrophic diseases estimated here was biased for WTI, because epidemics occurred not from the beginning of the study period onwards and were mainly episodic (Fig. 4). Starting with a rating of 1.0 to 1.7 for all biotrophic diseases in WTI in 1988, the breeding progress could be only very restricted (MLD in I2, YLR in I1 and I2) or even negative (MLD in I1, BNR in I1 and I2).

**MLD** can infect all cereals due to its different formae speciales. Nevertheless, it is one of the least important pathogens regarding its yield loss effects. This is mainly due to successful breeding by using quantitative resistances, at least in WW (Miedaner and Flath 2007). **MLD** susceptibility was considerably improved in the range of − 0.6 (WB 2r) to − 1.0 (WW), except in WTI and WR. During 1988–2019, the effect of fungicides reduced MLD infection nearly completely in all crops (conf. Figures 3, 4), even in WTI.

The same degree of control in I2 was also found for **BNR** in WW, WTI and WR. While susceptibility to BNR was strongly improved in WR Hyb by − 1.3 scores, it still maintains at a high average score of 4.1 in I1. The susceptibility level for **STB** is the highest compared with other diseases, and the application of fungicides is less effective. In WR Pop no breeding progress was found for MLD and BNR as might be explained by the low breeding activities since the upcoming of widely used hybrid varieties.

**YLR** occurred only episodically in wheat (1989–1990, 1999, 2001, 2014–2016) (Fig. 4) as in 1999 due to the breakdown of the gene *Yr17* (Bayles et al. 2000), but became increasingly important in the last decade due to the advent of the aggressive Warrior race in 2011 (Hovmøller et al. 2016). Reduction in susceptibility for YLR was low in WW (− 0.3) and WTI (− 0.1). The latter could be attributed to the change in susceptibility of some varieties according to the rapidly changing YLR races from the NW European population to the Warrior and Warrior (-) races (Miedaner and Juroszek 2021). However, this was the average change over 31 years, where varieties were mostly resistant against YLR until 2013. The epidemics of YLR led to an increased application of fungicides in WW (see Fig. 1) to such an extent that YLR could be controlled nearly completely during YLR epidemic years.

Susceptibility trend for **RYS** decreased in WR, WB and SB in I1 and I2. In recent years RYS I2 was nearly fully controlled in WB and SB, but not in WR, where the susceptibility level was notably higher compared to WB and SB (Table 3). Presumably, breeding activities did not focus on improving RYS susceptibility in WR hybrid varieties. In the WR population varieties, a remarkable breeding progress was detected for RYS in I1 and I2; however, only a low number of varieties was included in the analysis.

**NTB** decreased in both intensities in WB, but not in SB I1 where an increase of susceptibility by 0.5 was estimated. As shown in Figs. 3, 4, **DLR** was fully controlled in I2, but in I1 the susceptibility in WB 6r and SB decreased considerably until 2008 and then a steep increase followed until 2019. This u-shaped trend cannot be ascribed to changing sensitivity of varieties but should rather be attributed to a lower DLR infestation in the respective years.

Generally, stem stability decreased whereas disease susceptibility was improved for diseases in most crops, but not so for YLR in WW. The application of fungicides and growth regulators decreased LDG, but was less effective in reducing SBL and EBL. Fungicide application was very effective for most diseases and in most crops, except for STB in WW and RYS in WR. Figures 3a and 4 show very similar susceptibility pattern for WR Hyb and Pop varieties, except for BNR where WR Pop varieties were considerably less susceptible in I1 and I2 than WR Hyb varieties. WTI showed a trend pattern for most diseases which was different from the other crops due to the gradual adaptation of wheat and rye diseases to WTI during the study period.

Across all crops, considerable gaps between I2 and I1 in 1988 and 2017 are shown in Fig. 3 and Table 3, with most gaps widening. Decreasing gaps were found only for YLD, EAD and RYS in WW, WTI and WR. This widening of gaps indicated that progress in the treated intensity was higher than in the untreated, which does not mean that no breeding progress was achieved in I1. A stronger convergence between trends could have been expected because nitrogen application was reduced in I2 and increased in I1, while fungicide application level did not change much and only growth regulator application increased (Fig. 1).

### Trends for ageing effects of varieties

In Table 4 the changes due to ageing effects over 10 years were shown in I1, I2 and the difference in I2−I1. The sign of individual ageing effects depended on the intensity, traits and crops. Considerable ageing trends for yield and diseases have been reported, e.g. by Mackay et al. (2011) and Laidig et al. (2021) in WW variety trials. Both studies assumed that in the treated intensity no ageing effect was present, which means that genetic and non-genetic trends in I2 and I1 were identical. Further because there was no ageing trend in I2, the difference between I1 and I2 was an ageing effect of I1 on its own. Nevertheless, inspection of our data raised concern that this assumption may not hold true for all traits and all crops. Besides the ageing effect, we therefore additionally included a non-genetic time trend in Eqs. (3–10), and further considered intercepts of individual varieties as fixed to take into account genetic trends in reference varieties, i.e. that genetic trends are removed from trends of ageing effects.

Unfortunately, it was not possible to estimate both, trends of ageing effects and time trends separately, because they were linearly dependent (Piepho et al. 2014). Nevertheless, the results confirmed our approach; this could be demonstrated for HGT in WR Hyb and Pop, where ageing effects were found in I1 and I2, but not in I2−I1. It was unlikely that HGT of varieties were altered with increasing variety age, but other factors could influence HGT, e.g. trends of treatment intensity (Fig. 1), pre-cropping or soil tillage (Supplementary Material SM2, Fig. S1). The result for HGT in WR suggested that time trends were similar in I2 and I1 such that the ageing effect I2−I1 was not significant. For LDG the trends for ageing effects in crops given in Table 4 might be attributed to other factors. As such, we found a significant decrease of stem stability in all crops in I1, but not in I2. This stability of LDG I2, independent of variety age, could be ascribed to the increasing application rate of growth regulators as depicted in Fig. 1. In another case, e.g. MLD and BNR in WTI, ageing trends I2−I1 were underestimated, because in I2 an increasing susceptibility of varieties was estimated (*d*_2_ = 0.56 and *d*_1_ = 2.48 for MLD, *d*_1_ = 1.35 and *d*_2_ = 0.14 for BNR), resulting in reduced *d*_21_ =  − 2.03 for MLD and − 1.23 for BNR.

The largest trend in ageing effects were observed for BNR in WW (*d*_21_ =  − 1.31) and WTI (*d*_21_ =  − 1.24) (except for MLD (*d*_21_ =  − 2.03) in WTI). Concerning climate change, all future scenarios predict a further increase in BNR incidence and severity in NW Europe due to the thermophilic nature of the pathogen (Miedaner and Juroszek 2021). This is likely going to aggravate the existing BNR challenges and newly registered varieties may become susceptible to BNR more quickly, even leading to increasing ageing effects in future.

A further example for increased susceptibility of varieties was that WW pathogens adapted and became visibly virulent in the VCU trials of WTI from the years 1999 and 2001 onward for BNR and MLD, respectively (Figs. 3, 4). The same but inverse effect occurred in WB 6r for RYS; in this case susceptibility I1 (*d*_1_ =  − 0.22) and I2 (*d*_2_ =  − 0.25) decreased significantly resulting in non-significant I2−I1 (*d*_21_ =  − 0.07). We found no sound reason to explain the decreasing susceptibility in I1 and I2. We assume that this effect could be ascribed to other factors which acted in the same direction and about the same magnitude in both intensities.

As we have seen from the above, considerable ageing effects of different magnitude, direction and causes were present. Not only increasing sensitivity or even loss of resistance might be responsible for ageing effects, but also other factors might be involved like changing treatment intensities, altered management practices, climatic changes, or decrease in atmospheric nitrogen and sulfur dioxide deposition (Storkey et al. 2015; Chandramohan and Shaw 2013). Hence, to ensure that reference varieties represent the actual state of breeding progress, it is reasonable to replace reference varieties with newer ones not only to keep pace with breeding progress, but also to avoid biased trends due to increasing ageing effects.

### Genotypic and environmental variation

We estimated variance components removing genetic and non-genetic trends and assumed homogeneity, i.e. that components do not change over time. However, this assumption might not fully apply. Hadasch et al. (2020) showed that time trends of variance components were present in WW and WR variety trials, however, of lower magnitude indicating that increasing climatic conditions were likely to influence environmental variability of trials over time.

The large share of environmental variation of 80% for continuous and 62% for sore traits demonstrated the wide range of pedo-climatic conditions under which the trials in this study were carried out. However, the overall genotype and the genotype-environment variation was relatively low and accounted for only about 10% over all crops and traits. A low genotypic variation was estimated for YLD, which accounted for only 2% compared to 9% for score traits over all crops (see Supplementary Material SM4, Table S2). This means that varieties differed only slightly in yield potential, the most important trait. Only genotypes with high yield performance were finally released leading to less variation in this trait. The larger genotypic variation in WW compared to other cereals could mainly be explained by the different yield levels of quality groups (Laidig et al. 2017a). Another reason for the larger genotypic variation of score traits, compared to YLD, was likely due to the fact that only trials with visible disease infection were included which may have led to increased differentiation between genotypes. Additionally, selection intensity is usually much higher for YLD than for disease resistances, thus providing still susceptible genotypes in the VCU trials but not low yielding ones. This restricts the variance for YLD. As heritability estimates are usually higher for disease resistances than for YLD, we can conclude that the latter has a much more complex genetic architecture.

More environmental variation was explained for continuous traits than for score traits. However, we found twice as high residual variation in score traits, which indicates that scores were subject to larger assessment errors than continuous traits.

Figure 4 depicted a very large variation of TSv within years, because disease infestation was conditional on the presence of inoculum and the fulfillment of disease specific micro-climatic conditions, i.e. temperature and moisture requirements (Caubel et al. 2017). The concurrent presence of inoculum and fulfilment of climatic requirements were very likely to differ much stronger between trials than between years. These conditions also interacted much stronger with varieties, compared to conditions responsible for yield formation and interaction between varieties as shown by Fig. 3.

### Limitations of this study

The disease and stem stability traits were assessed by visual observations on an ordinal 1-to-9 scale, which is an appropriate and widely used method in plant breeding and official variety trials (Zhang et al. 2007). For diseases, this roughly follows a logarithmic transformation of the underlying percentage area of diseased leaves or spikelets (Bundessortenamt 2000, Sect. 4.1). In fact, not every field trial showed an effect on stem stability or incidence for a specific disease, i.e. there were quite some trials in which all varieties had a score of 1. For the assessment of trends of a specific disease, we included only those trials for which a severity score for this particular trait was 2 or higher. We did so, because the susceptibility of a variety could only be investigated in trials with visible symptoms. As such, the investigated score traits differed regarding their frequency of occurrence as shown in Fig. 2. For LDG in WR about 80% of trials showed lodging, where for YLR in WTI only 20% were diseased.

In this paper, we analysed ordinal scores on a 1-to-9 scale as if they were metric data. This approach clearly constituted an approximation as the assumptions underlying our linear mixed models could not strictly be met. In a previous study (Laidig et al. 2021), diagnostic residual plots for six WW diseases indicated, however, that no gross departures from assumptions of normality and homogeneity of variance were observed. Thus, we believed that our results were based on the best possible analyses, given the nature of the data. There were, of course, several dedicated statistical methods for ordinal data as alternatives to our approach (Breslow and Lin 1995; Shah and Madden 2004; McCullagh and Nelder 1989, § 9.2.4; Thöni 1985). As outlined in Laidig et al. (2021), however, none of the seemingly obvious alternative routes of analysis were viable options for our data. All of them required a larger sample of independent and identically distributed observations per treatment (variety-by-environment combination), and we did not have such data.

We evaluated breeding progress based on variety means using Eq. (2) and did not estimate variety means (genetic trend) by least square means eliminating year effects, because variety means based on scores may be biased downwards due to existing ageing effects (Piepho et al. 2014). For score traits, which are bounded below at 1, this ageing effect, combined with the bounded range, might result in least square estimates below 1 or in even negative scores, which are not interpretable (Laidig et al. 2021). In the previous section “Effect of variety ageing” we showed that considerable ageing effects occurred for reference varieties, e.g. in WTI for MLD and BNR, in WW for BNR. We therefore used only the first three trial years of reference varieties to eliminate the ageing effects possibly occurring in later years. A further complication in estimating trends by Eq. (2) might occur because variety means with successive first trial years were autocorrelated. Variety means of successive first trial years had two main effects from two trial years in common and varieties whose first trial year was two years different had one trial year in common. We therefore included an autocorrelation of first order AR(1) in Eq. (2). The estimated changes were often either not interpretable or not estimable, especially if autocorrelation was close to 0 or 1. We therefore did not apply the AR(1) option as Piepho et al. (2015) recommended not using AR(1) models in such cases as we found them. Year effects in variety means were not completely eliminated as in least square estimates, where year was a fixed factor, but years and trials within years were averaged and confounded with the genotypic effect.

Overall, due to the huge extent of the data, respective analysis and results, we were not able to go into such details as would be possible in individual crop-by-crop studies. Instead, in this study, we provided a comprehensive overview on the general breeding progress and ageing effects of cereal crops with regard to yield-related and disease resistance traits. Distinct follow-up studies focusing on single crops including plant physiological or rather agronomic aspects are planned.

## Conclusions

In this study we investigated major traits relevant for variety registration and information of growers regarding variety choice in cereal crops. For the first time, results of long-term trends were quantified and compared between five cereal crops based on genotypes grown under two different treatment intensities in a wide range of pedo-climatic conditions.

Considerable increasing yield trends was found suggesting very successful breeding progress in cereal crops, where the highest gains were achieved in winter barley and the lowest one in winter rye for population varieties. Yield gaps between treated and untreated intensity (without fungicides and growth regulators) increased from 1988 to 2017 in the barleys, but decreased in other cereals.

In contrast to increasing yield trends, however, stem stability decreased in all crops during 1988 and 2017 despite successful breeding activities for reduced plant height, except in winter rye. Hence, breeding towards higher stem stability requires more attention in the future. With the exception of rye, the potential of further reducing plant height without impairing further yield increases appears to have been exhausted in cereal crops. Our results demonstrated progress in improving disease resistances of varieties to mildew, brown (leaf) rust, scald and dwarf leaf rust but no or only a low increase for resistances to Septoria leaf blotch, yellow rust and net blotch. For stem stability and disease resistances, the declining trends in the treated intensity were stronger than in the untreated.

Our results revealed considerable trends due to ageing effects in yield, stem stability and diseases not only in the untreated but also in the treated intensity. The strongest decline for yield occurred in winter wheat and winter triticale. This decline was mainly attributed to a gradual loss of disease resistance and decreasing stem stability. The strongest increase in disease susceptibility due to ageing was shown for brown rust, followed by yellow rust and Septoria leaf blotch. Our results strongly demonstrated that evident ageing trends are calling for continued breeding efforts and release of new improved varieties to maintain performance at the same level.

The very large environmental variation of about 80% for yield and 60% for stem stability and diseases compared to genotypic variation of 2% and 9%, respectively, indicated the wide range of environmental and pedo-climatic conditions included in our study. For all crops, the year-to-year variation in yield was about four times that of stem stability and diseases, while the residual variation in yield was only half that of stem stability and diseases, suggesting that score traits are subject to larger assessment errors especially when the average infection severity is low.

This study gives important and novel evidence on the long-term breeding progress achieved, and on the performance-reducing ageing effects in all relevant cereal traits. The results and conclusions can therefore provide evidence supporting the ongoing discussion on the appropriateness of chemical plant protection in cereal crop management. Further intensification of resistance, breeding efforts will be necessary in the future in order to maintain the productivity of cereals with lower crop protection intensity and availability.

## Supplementary Information

Below is the link to the electronic supplementary material.

Supplementary file1 (PDF 110 kb)

Supplementary file2 (PDF 171 kb)

Supplementary file3 (PDF 320 kb)

Supplementary file4 (PDF 100 kb)
