# Supplementary material for: Long-term breeding progress of yield, yield-related, and disease resistance traits in five cereal crops of German variety trials
Source: Theor Appl Genet. 2021 Oct 15;134(12):3805–27. doi: 10.1007/s00122-021-03929-5 (PMC8580907; doi:10.1007/s00122-021-03929-5)
Supplement: Supplementary file 1 — Supplementary file1 (PDF 110 kb) [file 122_2021_3929_MOESM1_ESM.pdf]

Supplementary Material SM1 Description of lodging and diseases in cereal crops

Among cereals, wheat and triticale suffer from most diseases followed by rye and barley. Although powdery mildew (*Blumeria graminis*) (MLD) can infect all cereals due to its different formae specialis, it nevertheless is one of the least important pathogens regarding its yield loss effects. This is mainly due to successful breeding by using quantitative resistances, at least in wheat (Miedaner and Flath 2007), and the increasingly adverse weather conditions for successful infestation of MLD, because powdery mildew needs a high humidity of 95-100% for becoming epidemic (Serfling et al. 2017).

In **wheat,** brown (leaf) rust caused by *Puccinia triticina* and Septoria leaf blotch caused by *Zymoseptoria tritici* are currently among the most important fungi (Miedaner 2018). Yellow/stripe rust caused by *Puccinia striiformis*, was in previous times only episodically a problem for wheat (Figueroa et al. 2018), but became increasingly important in the last decade due to the advent of the aggressive Warrior race from the Himalayan region in Europe (Hovmøller et al. 2016). The Warrior race (named *Pst*S7), took over the European yellow rust population within one year and has more virulence genes than previous European populations, while it also infects triticale. Also today, still descendants of the Warrior race (namely *PstS10*) are predominant in Europe (https://agro.au.dk/forskning/internationale-platforme/wheatrust/yellow-rust-tools-maps-and-charts/races-changes-across-years/, accessed 03.03.2021).

The situation of variety resistance for rusts in wheat and barley is similar. Mainly race-specific resistances are used that are, however, not durable in most instances (Serfling et al. 2017, Figueroa et al. 2018, Singh et al. 2019). Additionally, quantitative resistances are available that are non-race specific, conferring a reduced pathogen development but are prone to high environmental variation (Serfling et al. 2017). Similarly, for Septoria leaf blotch, 21 major genes have been mapped that contribute to qualitative resistance (Brown et al., 2015), but sources of quantitative resistance have also been identified (Risser et al. 2011).

In **triticale**, the same fungi as in wheat play a major role. The biotrophic fungi adapted to this man-made cereal (Oettler 2005) during the last decades either by hybridization (e.g. powdery mildew, (Menardo et al. 2016) or by host jump (e.g. leaf rust, Visser et al. 2012). It must be noted, that these diseases of triticale were not available during the whole period of this investigation. Powdery mildew firstly appeared 2001 in Germany (Klocke et al. 2013) and in Belgium one year later (Audenaert et al. 2014). Similarly, the first infections with yellow rust occurred in 2001 and with leaf rust already in 1998 (Audenaert et al. 2014). Until that time, and for some time after, there was no resistance breeding against these diseases, because triticale was previously completely free of powdery mildew and rusts. For the necrotrophic *Z. tritici*, triticale was susceptible from the beginning (Fuentes 1973, Eyal and Talpaz 1990).

For **rye**, brown rust (*P. recondita*) and Rhynchosporium scald (*Rhynchosporium secalis*) are among the most important leaf diseases (Miedaner and Wilde, 2019). In brown rust, race-specific resistances are available that are, however, only of restricted durability because of the high virulence complexity of the leaf rust population in Germany (Miedaner et al. 2012).

In **winter and spring barley** powdery mildew (*B. graminis* f.sp. *hordei*), leaf rust, also called dwarf rust (*P. hordei*), net blotch (*Pyrenophora teres*) and Rhynchosporium scald (*R. commune*) are among the most prominent fungal diseases (Sing et al. 2019). For the two latter, race-specific resistances occur (Sing et al. 2019, Zhang et al. 2020a). Additionally, 148 QTLs from 34 studies were reported for scald resistance to date (Zhang et al. 2020a).

Despite the immense knowledge that has accumulated on disease resistance in cereals, the resistance situation in the current German variety spectrum and especially the development over the last decades is unclear.

Lodging occurs in all cereals; it is a complicated phenomenon that is influenced by many factors including: wind, rain, topography, soil type, previous crop, husbandry and disease (Berry et al. 2004). Lodging can partially be caused by fungal pathogens that attack the basal shoots already during early vegetative growth: eyespot caused by *Oculimacula spp*. in wheat and rye (Serfling et al. 2017), additionally *Microdochium nivale* and *Fusarium spp*. can play a role in all cereals (Miedaner 2018). Progress in lodging tolerance in winter wheat has been reported by Berry et al. 2004, Berry et al 2015 and Zhang et al. 2020b mainly in consequence of reduced plant height and improved application of growth regulators, while higher nitrogen application rates and increasing yield potential have raised the risk for lodging.

References

Audenaert, K, Troch V, Landschoot S, Haesaert G (2014) Biotic stresses in the anthropogenic hybrid triticale (× Triticosecale Wittmack): current knowledge and breeding challenges. European Journal of Plant Pathology, 140:615-630

Berry P M, Sterling M, Spink J H, Baker C J, Sylvester-Bradley R, Mooney S J, Tams A R, Ennos A R (2004) Understanding and reducing lodging in cereals. Advances in Agronomy, 84:215–269

Berry P M, Kendall S, Rutterford Z, Orford S, Griffiths S. (2015) Historical analysis of the effects of breeding on the height of winter wheat (Triticum aestivum) and consequences for lodging. Euphytica, 203:375–383

Brown JKM, Chartrain L, Lasserre-Zuber P, Saintenac C (2015) Genetics of resistance to Zymoseptoria tritici and applications to wheat breeding. Fungal Genet. Biol. 79:33–41. doi: 10.1016/j.fgb.2015.04.017

Eyal Z, Talpaz H (1990) The combined effect of plant stature and maturity on the response of wheat and triticale accessions to Septoria tritici. Euphytica 46:133-141

Figueroa M, Hammond-Kosack, K E, Solomon P. (2018) A review of wheat diseases - a field perspective. Molecular Plant Pathology 19: 1523-1536

Fuentes, FS (1973) Triticale diseases. Triticale breeding and research at CIMMYT. CIMMYT Research Bulletin 24:34-38 https://repository.cimmyt.org/bitstream/handle/10883/19412/25343.pdf?sequence=1#page=36 accessed 03.03.2021

Klocke B, Flath K, Miedaner T (2013) Virulence phenotypes in powdery mildew (Blumeria graminis) populations and resistance genes in triticale (x Triticosecale). Eur. J. Plant Pathol 137:463-476 Doi: 10.1007/s10658-013-0257-9.

Menardo, F, Praz CR, Wyder S, Ben-David R, Bourras S, Matsumae H, ... Keller B (2016) Hybridization of powdery mildew strains gives rise to pathogens on novel agricultural crop species. Nature Genetics 48:201-205

Miedaner T, Klocke B, Flath K, Geiger HH, Weber WE (2012) Diversity, spatial variation, and temporal dynamics of virulences in the German leaf rust (Puccinia recondita f. sp. secalis) population in winter rye. Eur. J. Plant Pathol 132:23-35. Doi: 10.1007/s10658-011-9845-8.

Miedaner, T. and K. Flath (2007) Effectiveness and environmental stability of quantitative powdery mildew (Blumeria graminis) resistance among winter wheat cultivars Plant Breed 126:553-558

Miedaner (Hrsg.) (2018). Management von Pilzkrankheiten im Ackerbau. Erling Verlag, Clenze, ISBN 978-3-86263-134-6

Miedaner T, Wilde P (2019) Selection strategies in hybrid rye with special consideration of fungal disease resistances. In Advances in breeding techniques for cereal crops. Burleigh Dodds Science Publishing. DOI: 10.19103/AS.2019.0051.12

Miedaner T, Hübner M, Korzun V, Schmiedchen B, Bauer E, Haseneyer G, Wilde P, Reif JC (2018) Genetic architecture of complex agronomic traits examined in two testcross populations of rye (Secale cereale L.). BMC Genomics. 13:706. doi: 10.1186/1471-2164-13-706

Oettler G (2005) The fortune of a botanical curiosity--Triticale: past, present and future. The Journal of Agricultural Science 143:329-346

Risser P, Ebmeyer E, Korzun V, Hartl L, Miedaner T (2011) Quantitative trait loci for adult-plant resistance to Mycosphaerella graminicola in two winter wheat populations. Phytopathology 101: 1209-1216. Doi: 10.1094/PHYTO-08-10-0203.

Serfling A, Kopahnke D, Habekuss A, Novakazi F, Ordon, F (2017) Wheat diseases: an overview. In: Langridge, P. (ed). Achieving sustainable cultivation of wheat, vol 1: Breeding, quality traits, pests and diseases. Pp. 1-32. Burleigh Dodds Sci Publ Ltd. http://dx.doi.org/10.19103/AS.2016.0004.19

Singh B, Mehta S, Aggarwal S K, Tiwari M, Bhuyan SI, Bhatia S, Islam, M A (2019) Barley, disease resistance, and molecular breeding approaches. In Disease resistance in crop plants (pp. 261-299). Springer, Cham.

Visser, B, Herselman L, Bender CM, Pretorius ZA (2012) Microsatellite analysis of selected Puccinia triticina races in South Africa. Australasian Plant Pathology 41:165-171

Zhang X, Ovenden B, Milgate A (2020a) Recent insights into barley and Rhynchosporium commune interactions. Molecular Plant Pathology 21:1111-1128

Zhang H, Teng L, Liu H, Mai C, Yu G, Li H, Yu L, Meng L, Jian D, Yang L, Li H, Zhou Y (2020b) Genetic progress in stem lodging resistance of the dominant wheat cultivars adapted to Yellow-Huai River Valleys Winter Wheat Zone in China since 1964. Journal of Integrative Agriculture 19:438–448
