# Supplementary material for: Long-term breeding progress of yield, yield-related, and disease resistance traits in five cereal crops of German variety trials
Source: Theor Appl Genet. 2021 Oct 15;134(12):3805–27. doi: 10.1007/s00122-021-03929-5 (PMC8580907; doi:10.1007/s00122-021-03929-5)
Supplement: Supplementary file 2 — Supplementary file2 (PDF 171 kb) [file 122_2021_3929_MOESM2_ESM.pdf]

Supplementary Material SM2 Pedo-climatic conditions, pre-cropping and tillage

Fig. S1 a) shows that WTI and WR trials are located at lower altitudes compared to SB trials. The long-term average annual precipitation differed only slightly between crops. A similar pattern was shown for long-term average annual temperature with the exception that the mean in WR was slightly lower than for other crops. Differences between crops in soil quality were more distinct. WR trials were conducted on soils with the lowest average quality grade of about 45, whereas in WW the average value was about 65 points followed by WB, SB, and WTI.

In Fig. S1 b) we display the situation in the first 5-years (1988-1992) vs. the last 5-years (2015-2019) period to retrieve a distinct picture for specific developments. As such, we categorized previous crops in foliage crops, maize and cereals and found an increasing share of foliage crops and maize of about 8% on average. In WW, maize increased by around 5%, leaf crops did not change and cereals had the lowest share as previous crops of only about 20% of trials. In contrast, in WTI and WB, cereals were the previous crop in nearly 40% and nearly 50% of trials, respectively. In WB and SB, cereals accounted for even more than 50%. Overall, the share of maize was small in the recent 5-year period, ranging between 2% and 9% depending on the crop.

Furthermore, we categorized soil tillage of trials in "plough" and "ploughless" and again compared the first (1988-1992) and last 5-years (2015-2019) period. Fig. S2 b shows that ploughless tillage increased by more than four times between the first and the last 5-years period, especially for WW, where ploughless tillage increased from 10% to more than 40%.

a)

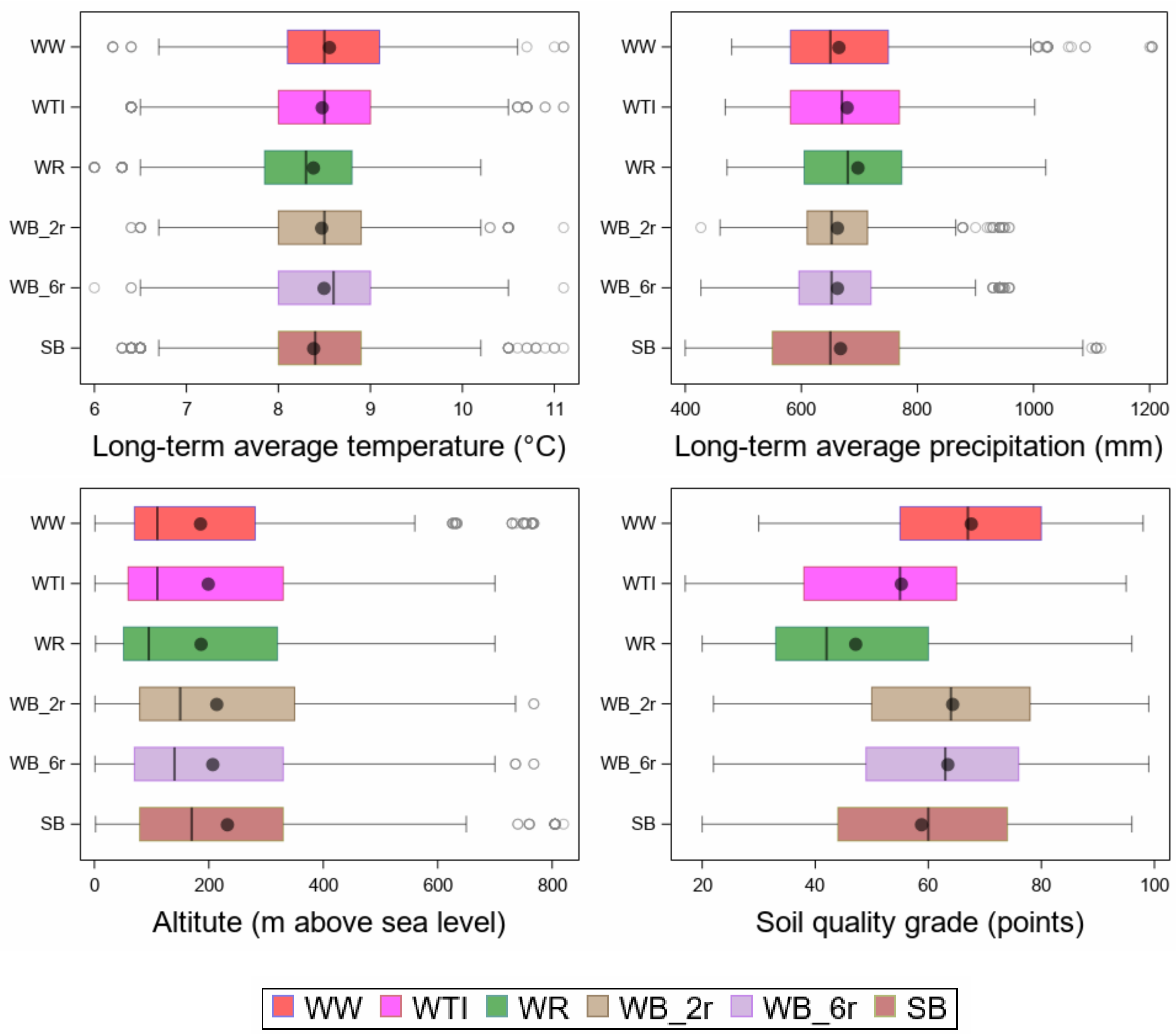

b)

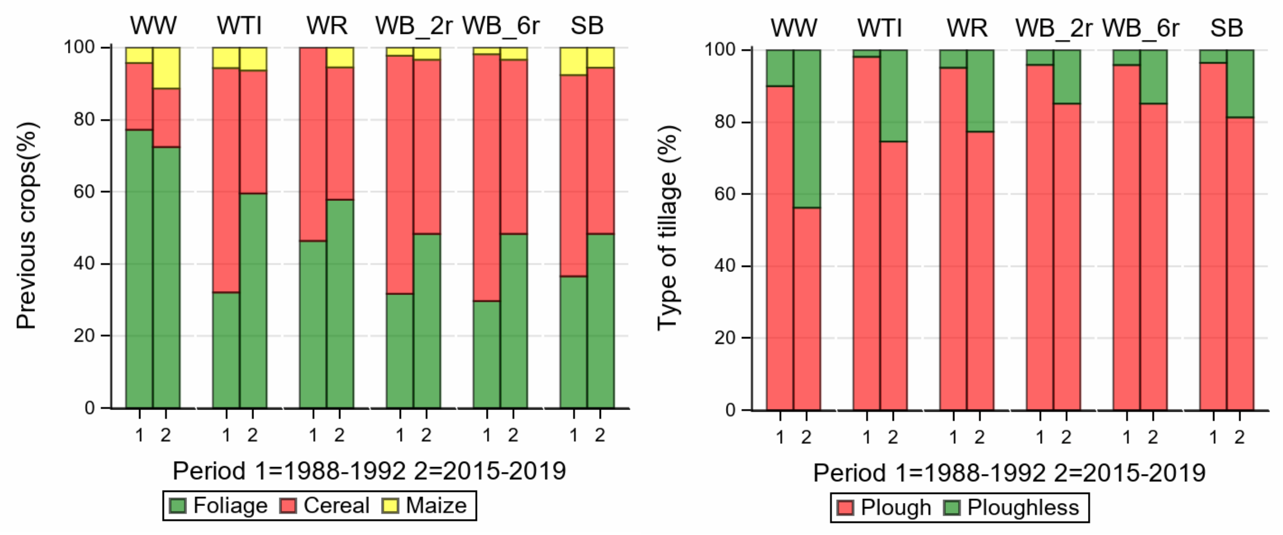

Fig. S1 a) Average pedo-climatic conditions 1988-2019 and **b)** pre-cropping and type of tillage between period 1 (1988-1992) and period 2 (2015-2019)

WW Winter wheat; *WTI* Winter triticale; *WR* Winter rye; *WB* Winter barley, *2r* two-row, *6r* six-row varieties; *SB* Spring barley
