# Supplementary material for: Long-term breeding progress of yield, yield-related, and disease resistance traits in five cereal crops of German variety trials
Source: Theor Appl Genet. 2021 Oct 15;134(12):3805–27. doi: 10.1007/s00122-021-03929-5 (PMC8580907; doi:10.1007/s00122-021-03929-5)
Supplement: Supplementary file 3 — Supplementary file3 (PDF 320 kb) [file 122_2021_3929_MOESM3_ESM.pdf]

Supplementary Material SM3 Effect of variety age over 10 years

Table S1 Change due to effect of variety age over 10 years by using Eqs. (3d) and (3f) for I1, I2 and I2-I1

d_1 represents the change of I1, d_2 of I2 and d_{21} of the difference I2 – I1.

δ_h Common regression coefficient for ageing trend, σ_h^2 Variance component for deviations of variety regression coefficients from the common coefficient. *SE* Standard error, *Probt* Significance of t-value.

WW Winter wheat; *WTI* Winter triticale; *WR* Winter rye, *Hyb* Hybrid, *Pop* Population varieties; *WB* Winter barley, *2r* two-row, *6r* six row varieties; *SB* Spring barley

YLD Yield (dt ha^{-2}); *EAD* Number of ears per m^2; *EAY* Single ear yield (g); *HGT* Plant height (cm); *EAE* Days from sowing to ear emergence; *LDG* Lodging; *SBL* Stem buckling; *EBL* Ear buckling; *MLD* Powdery mildew; *BNR* Brown rust; *STB* Septoria leaf blotch; *RYS* Rhynchosporium; *YLR* Yellow rust; *SNB* Septoria nodorum blotch; *NTB* Net blotch; *DLR* Dwarf leaf rust;

^{ns} non-significant; *Significant at 5% level; ** Significant at 1% level; *** Significant at 0.1% level;

Crop	Trait		Change		δ_h	SE	Probt	$\sqrt{\sigma_h^2}$
WW	YLD	d_1	-7.062	***	-0.7062	0.1466	0.0000	0.4197
WW	YLD	d_2	-4.473	**	-0.4473	0.1411	0.0015	0.4106
WW	YLD	d_{21}	2.840	*	0.2840	0.1131	0.0120	0.3838
WW	EAD	d_1	-1.958	ns	-0.1958	0.9289	0.8331	1.5423
WW	EAD	d_2	-5.524	ns	-0.5524	0.8769	0.5288	0.0000
WW	EAD	d_{21}	-6.411	ns	-0.6411	0.4176	0.1248	0.8354
WW	EAY	d_1	-0.128	***	-0.0128	0.0034	0.0002	0.0083
WW	EAY	d_2	-0.036	ns	-0.0036	0.0029	0.2219	0.0039
WW	EAY	d_{21}	0.079	**	0.0079	0.0024	0.0010	0.0071
WW	HGT	d_1	-0.247	ns	-0.0247	0.1027	0.8099	0.0549
WW	HGT	d_2	-1.513	ns	-0.1513	0.0986	0.1249	0.0900
WW	HGT	d_{21}	-1.345	***	-0.1345	0.0376	0.0003	0.0692
WW	LDG	d_1	0.334	*	0.0334	0.0170	0.0497	0.0067
WW	LDG	d_2	0.016	ns	0.0016	0.0136	0.9089	0.0045
WW	LDG	d_{21}	-0.345	***	-0.0345	0.0084	0.0000	0.0000
WW	MLD	d_1	0.278	*	0.0278	0.0135	0.0400	0.0468
WW	MLD	d_2	0.006	ns	0.0006	0.0060	0.9208	0.0190
WW	MLD	d_{21}	-0.234	*	-0.0234	0.0105	0.0257	0.0275
WW	BNR	d_1	1.295	***	0.1295	0.0275	0.0000	0.1235
WW	BNR	d_2	-0.011	ns	-0.0011	0.0050	0.8247	0.0033
WW	BNR	d_{21}	-1.309	***	-0.1309	0.0262	0.0000	0.1188
WW	STB	d_1	0.354	**	0.0354	0.0127	0.0052	0.0329
WW	STB	d_2	-0.044	ns	-0.0044	0.0077	0.5702	0.0167
WW	STB	d_{21}	-0.405	***	-0.0405	0.0098	0.0000	0.0187
WW	SNB	d_1	-0.093	ns	-0.0093	0.0123	0.4502	0.0158
WW	SNB	d_2	-0.230	**	-0.0230	0.0083	0.0054	0.0000
WW	SNB	d_{21}	-0.127	ns	-0.0127	0.0104	0.2215	0.0071
WW	YLR	d_1	0.741	*	0.0741	0.0312	0.0178	0.1331
WW	YLR	d_2	-0.010	ns	-0.0010	0.0073	0.8921	0.0244
WW	YLR	d_{21}	-0.778	**	-0.0778	0.0284	0.0062	0.1211
WTI	YLD	d_1	-10.268	***	-1.0268	0.2065	0.0000	0.6858
WTI	YLD	d_2	-4.681	**	-0.4681	0.1425	0.0010	0.0000
WTI	YLD	d_{21}	5.671	**	0.5671	0.2081	0.0064	0.9233
WTI	EAD	d_1	8.654	ns	0.8654	0.8958	0.3341	1.2658
WTI	EAD	d_2	9.045	ns	0.9045	0.8360	0.2793	0.2090
WTI	EAD	d_{21}	-0.399	ns	-0.0399	0.3705	0.9143	0.5914
WTI	EAY	d_1	-0.229	***	-0.0229	0.0037	0.0000	0.0077
WTI	EAY	d_2	-0.133	***	-0.0133	0.0032	0.0000	0.0000
WTI	EAY	d_{21}	0.094	**	0.0094	0.0034	0.0054	0.0109
WTI	HGT	d_1	0.648	ns	0.0648	0.1290	0.6155	0.1040

WTI	HGT	d_2	0.889	ns	0.0889	0.1415	0.5300	0.2237
WTI	HGT	d_{21}	0.431	ns	0.0431	0.0637	0.4987	0.1328
WTI	LDG	d_1	0.424	*	0.0424	0.0166	0.0104	0.0026
WTI	LDG	d_2	0.033	ns	0.0033	0.0129	0.7987	0.0000
WTI	LDG	d_{21}	-0.381	**	-0.0381	0.0134	0.0046	0.0147
WTI	MLD	d_1	2.476	***	0.2476	0.0486	0.0000	0.2240
WTI	MLD	d_2	0.560	***	0.0560	0.0157	0.0004	0.0677
WTI	MLD	d_{21}	-2.033	***	-0.2033	0.0378	0.0000	0.1681
WTI	BNR	d_1	1.353	***	0.1353	0.0285	0.0000	0.0998
WTI	BNR	d_2	0.139	***	0.0139	0.0031	0.0000	0.0000
WTI	BNR	d_{21}	-1.238	***	-0.1238	0.0275	0.0000	0.0963
WTI	STB	d_1	0.397	***	0.0397	0.0109	0.0003	0.0063
WTI	STB	d_2	-0.115	ns	-0.0115	0.0094	0.2212	0.0200
WTI	STB	d_{21}	-0.519	***	-0.0519	0.0081	0.0000	0.0000
WTI	YLR	d_1	1.050	*	0.1050	0.0474	0.0268	0.1783
WTI	YLR	d_2	-0.011	ns	-0.0011	0.0077	0.8882	0.0138
WTI	YLR	d_{21}	-1.106	*	-0.1106	0.0457	0.0156	0.1778
WR Hyb	YLD	d_1	-4.401	***	-0.4401	0.1302	0.0007	0.1574
WR Hyb	YLD	d_2	-4.485	**	-0.4485	0.1721	0.0092	0.3488
WR Hyb	YLD	d_{21}	0.204	ns	0.0204	0.0942	0.8284	0.1369
WR Hyb	EAD	d_1	9.575	ns	0.9575	1.0677	0.3699	1.3143
WR Hyb	EAD	d_2	2.584	ns	0.2584	1.0522	0.8060	1.1168
WR Hyb	EAD	d_{21}	-7.976	ns	-0.7976	0.5688	0.1609	0.0000
WR Hyb	EAD	d_1	9.575	ns	0.9575	1.0677	0.3699	1.3143
WR Hyb	EAD	d_2	2.584	ns	0.2584	1.0522	0.8060	1.1168
WR Hyb	EAD	d_{21}	-7.976	ns	-0.7976	0.5688	0.1609	0.0000
WR Hyb	HGT	d_1	4.793	**	0.4793	0.1483	0.0012	0.1477
WR Hyb	HGT	d_2	3.971	*	0.3971	0.1849	0.0318	0.2991
WR Hyb	HGT	d_{21}	-0.865	ns	-0.0865	0.0636	0.1740	0.0255
WR Hyb	LDG	d_1	0.562	**	0.0562	0.0200	0.0049	0.0029
WR Hyb	LDG	d_2	-0.049	ns	-0.0049	0.0210	0.8169	0.0377
WR Hyb	LDG	d_{21}	-0.571	***	-0.0571	0.0151	0.0002	0.0000
WR Hyb	SBL	d_1	0.382	*	0.0382	0.0188	0.0421	0.0000
WR Hyb	SBL	d_2	0.251	ns	0.0251	0.0174	0.1492	0.0288
WR Hyb	SBL	d_{21}	-0.138	ns	-0.0138	0.0122	0.2578	0.0000
WR Hyb	MLD	d_1	0.161	ns	0.0161	0.0191	0.3993	0.0121
WR Hyb	MLD	d_2	0.187	ns	0.0187	0.0118	0.1144	0.0146
WR Hyb	MLD	d_{21}	-0.099	ns	-0.0099	0.0215	0.6447	0.0390
WR Hyb	BNR	d_1	1.076	**	0.1076	0.0329	0.0011	0.1096
WR Hyb	BNR	d_2	0.155	ns	0.0155	0.0122	0.2035	0.0174
WR Hyb	BNR	d_{21}	-0.984	**	-0.0984	0.0303	0.0012	0.1065
WR Hyb	RYS	d_1	0.004	ns	0.0004	0.0103	0.9666	0.0000

WR Hyb	RYS	d_2	0.033	ns	0.0033	0.0073	0.6499	0.0000
WR Hyb	RYS	d_{21}	0.028	ns	0.0028	0.0095	0.7655	0.0040
WR Pop	YLD	d_1	-2.241	*	-0.2241	0.1004	0.0257	0.0000
WR Pop	YLD	d_2	-2.121	ns	-0.2121	0.1905	0.2657	0.3542
WR Pop	YLD	d_{21}	-0.181	ns	-0.0181	0.1517	0.9050	0.2929
WR Pop	EAD	d_1	-3.954	ns	-0.3954	1.1497	0.7310	1.7747
WR Pop	EAD	d_2	-9.458	ns	-0.9458	0.8238	0.2511	0.0000
WR Pop	EAD	d_{21}	-8.537	ns	-0.8537	0.6865	0.2138	0.8102
WR Pop	EAY	d_1	-0.042	ns	-0.0042	0.0028	0.1317	0.0000
WR Pop	EAY	d_2	-0.012	ns	-0.0012	0.0031	0.7098	0.0000
WR Pop	EAY	d_{21}	0.029	ns	0.0029	0.0035	0.4077	0.0057
WR Pop	HGT	d_1	8.446	***	0.8446	0.1968	0.0000	0.3111
WR Pop	HGT	d_2	6.210	**	0.6210	0.2141	0.0038	0.3212
WR Pop	HGT	d_{21}	-1.191	ns	-0.1191	0.0789	0.1311	0.0000
WR Pop	LDG	d_1	0.761	**	0.0761	0.0265	0.0042	0.0414
WR Pop	LDG	d_2	0.142	ns	0.0142	0.0201	0.4809	0.0209
WR Pop	LDG	d_{21}	-0.620	**	-0.0620	0.0201	0.0021	0.0286
WR Pop	SBL	d_1	0.348	ns	0.0348	0.0180	0.0533	0.0000
WR Pop	SBL	d_2	0.055	ns	0.0055	0.0167	0.7426	0.0031
WR Pop	SBL	d_{21}	-0.198	ns	-0.0198	0.0138	0.1535	0.0000
WR Pop	MLD	d_1	-0.157	ns	-0.0157	0.0165	0.3435	0.0000
WR Pop	MLD	d_2	-0.015	ns	-0.0015	0.0095	0.8761	0.0000
WR Pop	MLD	d_{21}	0.137	ns	0.0137	0.0161	0.3926	0.0000
WR Pop	BNR	d_1	0.383	*	0.0383	0.0159	0.0158	0.0114
WR Pop	BNR	d_2	-0.163	ns	-0.0163	0.0186	0.3817	0.0363
WR Pop	BNR	d_{21}	-0.588	ns	-0.0588	0.0335	0.0797	0.0761
WR Pop	RYS	d_1	0.056	ns	0.0056	0.0110	0.6128	0.0074
WR Pop	RYS	d_2	-0.142	ns	-0.0142	0.0084	0.0897	0.0000
WR Pop	RYS	d_{21}	-0.193	ns	-0.0193	0.0119	0.1048	0.0126
WB 2r	YLD	d_1	-0.877	ns	-0.0877	0.1026	0.3927	0.1374
WB 2r	YLD	d_2	0.901	ns	0.0901	0.1109	0.4166	0.0000
WB 2r	YLD	d_{21}	1.418	*	0.1418	0.0674	0.0354	0.0000
WB 2r	EAD	d_1	19.235	ns	1.9235	1.7519	0.2723	2.2036
WB 2r	EAD	d_2	18.697	ns	1.8697	1.7531	0.2862	2.5223
WB 2r	EAD	d_{21}	3.821	ns	0.3821	0.7008	0.5856	0.0000
WB 2r	EAY	d_1	-0.030	ns	-0.0030	0.0018	0.0994	0.0026
WB 2r	EAY	d_2	-0.023	ns	-0.0023	0.0018	0.2108	0.0043
WB 2r	EAY	d_{21}	0.006	ns	0.0006	0.0010	0.5362	0.0000
WB 2r	HGT	d_1	-0.176	ns	-0.0176	0.1270	0.8900	0.0530
WB 2r	HGT	d_2	-2.000	ns	-0.2000	0.1268	0.1148	0.1036
WB 2r	HGT	d_{21}	-1.661	***	-0.1661	0.0415	0.0001	0.0000
WB 2r	LDG	d_1	0.407	*	0.0407	0.0178	0.0224	0.0000

WB 2r	LDG	d_2	-0.100	ns	-0.0100	0.0166	0.5472	0.0321
WB 2r	LDG	d_{21}	-0.507	***	-0.0507	0.0131	0.0001	0.0000
WB 2r	SBL	d_1	0.704	***	0.0704	0.0186	0.0002	0.0218
WB 2r	SBL	d_2	0.160	ns	0.0160	0.0119	0.1796	0.0148
WB 2r	SBL	d_{21}	-0.521	***	-0.0521	0.0126	0.0000	0.0000
WB 2r	EBL	d_1	0.271	*	0.0271	0.0116	0.0195	0.0000
WB 2r	EBL	d_2	0.116	ns	0.0116	0.0111	0.2946	0.0000
WB 2r	EBL	d_{21}	-0.176	*	-0.0176	0.0087	0.0420	0.0165
WB 2r	MLD	d_1	-0.299	ns	-0.0299	0.0160	0.0612	0.0350
WB 2r	MLD	d_2	-0.224	**	-0.0224	0.0083	0.0068	0.0098
WB 2r	MLD	d_{21}	0.114	ns	0.0114	0.0127	0.3670	0.0136
WB 2r	RYS	d_1	0.395	*	0.0395	0.0185	0.0328	0.0697
WB 2r	RYS	d_2	-0.095	ns	-0.0095	0.0083	0.2512	0.0232
WB 2r	RYS	d_{21}	-0.475	**	-0.0475	0.0150	0.0016	0.0568
WB 2r	NTB	d_1	0.058	ns	0.0058	0.0105	0.5822	0.0122
WB 2r	NTB	d_2	-0.114	ns	-0.0114	0.0062	0.0678	0.0121
WB 2r	NTB	d_{21}	-0.150	ns	-0.0150	0.0101	0.1362	0.0260
WB 2r	DLR	d_1	0.150	ns	0.0150	0.0157	0.3385	0.0155
WB 2r	DLR	d_2	-0.096	*	-0.0096	0.0047	0.0387	0.0000
WB 2r	DLR	d_{21}	-0.243	ns	-0.0243	0.0137	0.0772	0.0067
WB 6r	YLD	d_1	-3.134	*	-0.3134	0.1269	0.0135	0.2832
WB 6r	YLD	d_2	-0.751	ns	-0.0751	0.1260	0.5515	0.1962
WB 6r	YLD	d_{21}	2.121	**	0.2121	0.0686	0.0020	0.0678
WB 6r	EAD	d_1	21.964	*	2.1964	1.1119	0.0483	1.3586
WB 6r	EAD	d_2	15.268	ns	1.5268	1.1497	0.1842	1.6345
WB 6r	EAD	d_{21}	-6.955	ns	-0.6955	0.4686	0.1377	0.0000
WB 6r	EAY	d_1	-0.111	***	-0.0111	0.0022	0.0000	0.0000
WB 6r	EAY	d_2	-0.067	**	-0.0067	0.0023	0.0031	0.0000
WB 6r	EAY	d_{21}	0.047	**	0.0047	0.0016	0.0037	0.0017
WB 6r	HGT	d_1	1.989	ns	0.1989	0.1316	0.1308	0.0000
WB 6r	HGT	d_2	1.068	ns	0.1068	0.1352	0.4295	0.0000
WB 6r	HGT	d_{21}	-1.062	**	-0.1062	0.0382	0.0055	0.0000
WB 6r	LDG	d_1	0.541	***	0.0541	0.0161	0.0008	0.0000
WB 6r	LDG	d_2	-0.017	ns	-0.0017	0.0151	0.9088	0.0117
WB 6r	LDG	d_{21}	-0.508	***	-0.0508	0.0153	0.0009	0.0221
WB 6r	SBL	d_1	0.960	***	0.0960	0.0192	0.0000	0.0421
WB 6r	SBL	d_2	0.350	*	0.0350	0.0138	0.0113	0.0260
WB 6r	SBL	d_{21}	-0.551	***	-0.0551	0.0124	0.0000	0.0000
WB 6r	EBL	d_1	0.250	ns	0.0250	0.0144	0.0829	0.0000
WB 6r	EBL	d_2	0.231	ns	0.0231	0.0147	0.1159	0.0135
WB 6r	EBL	d_{21}	0.047	ns	0.0047	0.0121	0.6977	0.0318
WB 6r	MLD	d_1	0.570	ns	0.0570	0.0312	0.0682	0.1445

WB 6r	MLD	d_2	-0.136	*	-0.0136	0.0058	0.0187	0.0122
WB 6r	MLD	d_{21}	-0.693	*	-0.0693	0.0286	0.0156	0.1329
WB 6r	RYS	d_1	-0.216	*	-0.0216	0.0095	0.0226	0.0108
WB 6r	RYS	d_2	-0.252	***	-0.0252	0.0066	0.0001	0.0147
WB 6r	RYS	d_{21}	-0.067	ns	-0.0067	0.0074	0.3623	0.0000
WB 6r	NTB	d_1	0.547	***	0.0547	0.0148	0.0002	0.0452
WB 6r	NTB	d_2	-0.071	ns	-0.0071	0.0052	0.1694	0.0000
WB 6r	NTB	d_{21}	-0.662	***	-0.0662	0.0125	0.0000	0.0426
WB 6r	DLR	d_1	0.344	ns	0.0344	0.0268	0.1993	0.1017
WB 6r	DLR	d_2	-0.231	***	-0.0231	0.0050	0.0000	0.0056
WB 6r	DLR	d_{21}	-0.497	*	-0.0497	0.0220	0.0241	0.0747
SB	YLD	d_1	-3.252	**	-0.3252	0.1195	0.0065	0.2082
SB	YLD	d_2	-1.852	ns	-0.1852	0.1192	0.1203	0.1436
SB	YLD	d_{21}	1.046	*	0.1046	0.0490	0.0326	0.0859
SB	EAD	d_1	7.373	ns	0.7373	1.0245	0.4717	0.6652
SB	EAD	d_2	-6.160	ns	-0.6160	1.1526	0.5931	2.1200
SB	EAD	d_{21}	-12.775	*	-1.2775	0.5947	0.0317	1.1718
SB	EAY	d_1	-0.046	**	-0.0046	0.0016	0.0045	0.0013
SB	EAY	d_2	-0.029	ns	-0.0029	0.0017	0.0903	0.0035
SB	EAY	d_{21}	0.021	**	0.0021	0.0008	0.0080	0.0012
SB	HGT	d_1	1.387	ns	0.1387	0.0898	0.1226	0.0700
SB	HGT	d_2	1.151	ns	0.1151	0.0837	0.1690	0.0445
SB	HGT	d_{21}	-0.366	ns	-0.0366	0.0316	0.2467	0.0228
SB	LDG	d_1	0.550	***	0.0550	0.0159	0.0006	0.0101
SB	LDG	d_2	0.149	ns	0.0149	0.0156	0.3417	0.0330
SB	LDG	d_{21}	-0.483	***	-0.0483	0.0128	0.0002	0.0327
SB	SBL	d_1	0.612	***	0.0612	0.0165	0.0002	0.0346
SB	SBL	d_2	0.459	**	0.0459	0.0140	0.0011	0.0409
SB	SBL	d_{21}	-0.229	*	-0.0229	0.0102	0.0246	0.0169
SB	EBL	d_1	0.428	**	0.0428	0.0147	0.0036	0.0379
SB	EBL	d_2	0.352	**	0.0352	0.0115	0.0022	0.0133
SB	EBL	d_{21}	-0.180	**	-0.0180	0.0056	0.0014	0.0000
SB	MLD	d_1	0.609	*	0.0609	0.0273	0.0257	0.1286
SB	MLD	d_2	0.137	ns	0.0137	0.0101	0.1758	0.0384
SB	MLD	d_{21}	-0.529	**	-0.0529	0.0200	0.0083	0.0914
SB	RYS	d_1	-0.123	ns	-0.0123	0.0127	0.3300	0.0180
SB	RYS	d_2	-0.143	ns	-0.0143	0.0081	0.0794	0.0124
SB	RYS	d_{21}	-0.018	ns	-0.0018	0.0082	0.8303	0.0000
SB	NTB	d_1	0.227	*	0.0227	0.0110	0.0392	0.0268
SB	NTB	d_2	-0.115	*	-0.0115	0.0058	0.0461	0.0083
SB	NTB	d_{21}	-0.290	***	-0.0290	0.0072	0.0001	0.0000
SB	DLR	d_1	0.291	ns	0.0291	0.0198	0.1421	0.0702

SB	DLR	d_2	-0.062	ns	-0.0062	0.0049	0.1994	0.0000
SB	DLR	d_{21}	-0.370	*	-0.0370	0.0171	0.0308	0.0547
