# Supplementary material for: Long-term breeding progress of yield, yield-related, and disease resistance traits in five cereal crops of German variety trials
Source: Theor Appl Genet. 2021 Oct 15;134(12):3805–27. doi: 10.1007/s00122-021-03929-5 (PMC8580907; doi:10.1007/s00122-021-03929-5)
Supplement: Supplementary file 4 — Supplementary file4 (PDF 100 kb) [file 122_2021_3929_MOESM4_ESM.pdf]

Supplementary Material SM4 Genotypic and environmental variation

Table S2 Variance components for a) continuous and b) score traits in intensity 1 as percent of total sum of components using basic model given by Eq. (1) assuming a non-linear trend in the genotype effect G_i (Eq. (4)) and linear trend in the year effect Y_k (Eq. (5)).

**Crop**	**WW**					**WTI**					**WR Hyb**					**WR Pop**					**Mean**
**Var**	YLD	EAD	EAY	HGT	EAE	YLD	EAD	EAY	HGT	EAE	*YLD*	EAD	EAY	HGT	EAE	YLD	EAD	EAY	HGT	EAE	
**G**	7	7	7	43	2	2	6	6	31	2	1	3	3	7	0	*1*	2	1	10	0	**6**
**Y**	11	8	8	15	24	9	6	5	13	15	9	4	5	16	20	*9*	4	6	15	22	**12**
**L**	18	19	16	8	46	28	30	17	23	56	29	39	24	24	42	*24*	46	24	25	42	**28**
**YxL**	46	41	42	25	27	41	38	44	24	25	49	35	45	46	36	*55*	31	46	44	35	**40**
**GxY**	3	1	1	1	0	3	1	2	1	0	1	1	0	1	0	1	0	1	1	0	**1**
**GxL**	2	1	1	1	0	3	1	3	1	0	1	0	0	0	0	1	1	1	0	0	**1**
**Resid**	13	24	25	7	1	14	19	24	6	1	9	18	21	6	1	9	16	21	6	1	**12**

**Crop**	**WB 2r**					**WB 6r**					**SB**					**Mean**
**Var**	YLD	EAD	EAY	HGT	EAE	YLD	EAD	EAY	HGT	EAE	YLD	EAD	EAY	HGT	EAE	
**G**	2	8	7	10	3	1	4	4	9	2	2	5	3	11	1	**6**
**Y**	9	9	6	17	29	10	10	5	18	28	11	3	7	8	35	**12**
**L**	20	22	19	25	42	25	23	20	24	45	27	22	24	27	20	**28**
**YxL**	54	39	44	41	23	48	39	42	41	23	49	49	45	45	41	**40**
**GxY**	2	1	1	1	1	2	1	1	1	0	1	1	1	1	0	**1**
**GxL**	1	1	1	0	0	1	0	0	0	0	1	1	1	0	0	**1**
**Resid**	12	20	22	7	2	13	23	28	6	2	8	20	20	8	2	**12**

**Crop**	**WW**						**WTI**					**WR Hyb**					**WR Pop**					**Mean**
**Var**	LDG	MLD	BNR	STB	SNB	YLR	LDG	SBL	MLD	BNR	RYS	LDG	SBL	MLD	BNR	RYS	LDG	MLD	BNR	STB	YLR	
**G**	9	15	15	6	6	14	12	16	18	4	10	4	4	7	3	2	7	4	5	10	2	**9**
**Y**	5	3	7	2	0	5	2	5	1	1	7	7	5	6	10	1	5	5	6	7	1	**4**
**L**	7	20	10	14	27	10	5	8	5	14	4	14	35	16	15	22	14	37	18	17	25	**15**
**YxL**	49	30	30	48	36	23	51	24	24	50	32	57	36	50	54	58	56	39	50	50	58	**43**
**GxY**	2	2	4	2	1	6	2	10	8	2	7	1	2	2	2	1	1	1	0	1	0	**2**
**GxL**	2	4	4	4	8	8	3	5	4	3	8	1	2	2	2	1	1	1	1	1	1	**3**
**Resid**	26	27	29	23	22	34	26	31	39	27	33	16	17	17	14	16	15	14	19	14	13	**23**

**Crop**							**WB 2r**							**WB 6r**							**SB**	**Mean**
**Var**	LDG	SBL	EBL	MLD	NTB	RHY	DLR	LDG	SBL	EBL	MLD	NTB	RHY	DLR	LDG	SBL	EBL	MLD	NTB	RHY	DLR	
**G**	5	10	12	14	3	11	10	4	10	12	10	5	8	14	8	8	7	19	3	5	9	**9**
**Y**	5	8	4	3	2	5	4	2	5	6	4	2	3	11	2	7	5	1	0	4	1	**4**
**L**	9	12	14	16	17	18	10	12	12	16	17	14	19	7	8	11	32	12	23	13	18	**15**
**YxL**	59	45	37	35	50	33	46	62	45	35	36	47	39	37	57	47	33	21	49	50	45	**43**
**GxY**	1	2	2	1	1	2	2	1	2	2	3	2	1	2	1	1	1	6	1	1	2	**2**
**GxL**	1	2	5	5	3	4	4	1	3	4	4	5	3	4	0	2	5	9	3	2	4	**3**
**Resid**	20	21	26	25	24	27	24	18	24	25	25	25	26	25	23	23	18	33	22	25	21	**23**

G Genotype; *Y* Year; *L* Location; *YxL*: Year by location; *GxY*, *GxL*: Genotype by year, location; *Res* Residual
WW Winter wheat, *WTI* Winter triticale; *WR* Winter rye, *Hyb* Hybrid, *Pop* Population varieties; *WB* Winter barley, *2r* two-row, *6r* six row-varieties; *SB* Spring barley
YLD Grain yield; *EAR* Ear density; *EAY* Ear yield; *HGT* Plant height; *EAE* Days to ear emergence; *LDG* Lodging; *SBL* Stem buckling; *EBL* Ear buckling; *MLD* Powdery mildew; *BNR* Brown rust; *STB* Septoria leaf blotch; *RYS* Rhynchosporium; *YLR* Yellow rust; *SNB* Septoria nodorum blotch; *NTB* Net blotch; *DLR* Dwarf leaf rust;
